# Dynamics of p53 and NF-κB regulation in response to DNA damage and identification of target proteins suitable for therapeutic intervention

**DOI:** 10.1186/1752-0509-6-125

**Published:** 2012-09-15

**Authors:** Rainer Poltz, Michael Naumann

**Affiliations:** 1 Institute of Experimental Internal Medicine, Otto von Guericke University, Leipziger Str. 44, Magdeburg, 39120, Germany

**Keywords:** Topoisomerase inhibitors, Signal transduction, Cell cycle arrest, Apoptosis, Cancer, Logical model

## Abstract

**Background:**

The genome is continuously attacked by a variety of agents that cause DNA damage. Recognition of DNA lesions activates the cellular DNA damage response (DDR), which comprises a network of signal transduction pathways to maintain genome integrity. In response to severe DNA damage, cells undergo apoptosis to avoid transformation into tumour cells, or alternatively, the cells enter permanent cell cycle arrest, called senescence. Most tumour cells have defects in pathways leading to DNA repair or apoptosis. In addition, apoptosis could be counteracted by nuclear factor kappa B (NF-κB), the main anti-apoptotic transcription factor in the DDR. Despite the high clinical relevance, the interplay of the DDR pathways is poorly understood. For therapeutic purposes DNA damage signalling processes are induced to induce apoptosis in tumour cells. However, the efficiency of radio- and chemotherapy is strongly hampered by cell survival pathways in tumour cells. In this study logical modelling was performed to facilitate understanding of the complexity of the signal transduction networks in the DDR and to provide cancer treatment options.

**Results:**

Our comprehensive discrete logical model provided new insights into the dynamics of the DDR in human epithelial tumours. We identified new mechanisms by which the cell regulates the dynamics of the activation of the tumour suppressor p53 and NF-κB. Simulating therapeutic intervention by agents causing DNA single-strand breaks (SSBs) or DNA double-strand breaks (DSBs) we identified candidate target proteins for sensitization of carcinomas to therapeutic intervention. Further, we enlightened the DDR in different genetic diseases, and by failure mode analysis we defined molecular defects putatively contributing to carcinogenesis.

**Conclusion:**

By logic modelling we identified candidate target proteins that could be suitable for radio- and chemotherapy, and contributes to the design of more effective therapies.

## Background

DNA damage is of profound biomedical interest, as this type of lesions largely contributes to cancerogenesis [1]. DNA damage is induced by environmental factors, like ionizing radiation [2], but also by intrinsic agents, like metabolically generated reactive oxygen species [3]. Damaged DNA becomes bound by so-called sensor proteins, like replication protein A (RPA) or a complex composed of meiotic recombination 11 (Mre11) - radiation 50 (Rad50) - nijmegen breakage syndrome 1 (Nbs1) (MRN). They trigger a complex network of signal transduction pathways designated as DDR [1]. The DDR causes temporal cell cycle arrest, if the level of DNA damage is low, so the cell can repair it [4]. In response to severe DNA damage, cells undergo apoptosis to avoid transformation into tumour cells [5]. Alternatively, the cells enter permanent cell cycle arrest, called senescence [6]. In presence of DNA damage, the tumour suppressor p53 plays a key role in the decision between survival and death of the cell. Activated p53 either induces cell cycle arrest or apoptosis, mainly by activation of distinct target genes [7]. As shown in biochemical and network modelling studies, p53 levels oscillate in response to DNA damage caused by ionizing radiation. The p53-dependent expression of wild-type p53-induced phosphatase 1 (Wip1) and murine double minute 2 (MDM2) mediates the oscillations [8]. Whereas Wip1 is essential for the generation of oscillations, MDM2 mediates their fine tuning [9]. The duration of the oscillations was proposed to determine, whether p53 acts pro-apoptotic or not [9]. However, apoptosis can be counteracted by activation of NF-κB, the main anti-apoptotic transcription factor in the DDR. Posttranslational modifications of NF-κB essential modulator (NEMO) exert an important role in the signal transduction that links DNA damage in the nucleus with activation of NF-κB in the cytoplasm [10]. Whether DSBs trigger stable oscillations of NF-κB on the level of single cells has not been shown.

SSBs and DSBs are the most lethal types of DNA damage [2]. They can be triggered by ionizing radiation or topoisomerase inhibitors, as therapeutically applied to eliminate tumour cells. Usually, higher proliferation rates of tumour cells render them more susceptible to DNA damage induced apoptosis than normal cells [11]. The efficiency of DNA damaging therapies can be potentiated by blocking cell survival pathways in tumour cells (sensitization) [12]. A strategy to sensitize tumours to DNA damaging agents is adjuvant abolishment of cycle arrest, resulting in necrosis or apoptosis-like cell death by mitotic catastrophy [13]. Other important sensitization targets are components that contribute to NF-κB activation, which otherwise often impedes efficient elimination of cancer cells [14].

However, most tumour cells have a defective DDR [11]. Such molecular defects due to mutations within tumours can be exploited to selectively sensitize tumours to therapy. Inhibitions that result in cell death only in combination with a molecular defect in targeted tumour cells would predominantly eliminate the tumour. Corresponding proteins are thus ideal drug targets [15]. Based on a network modelling approach following this strategy, inhibition targets that sensitize p53-deficient tumours to DNA damaging treatment were found [16].

Despite the high clinical relevance of the DDR, the interplay of the signal transduction involved herein is poorly understood, particularly due to high complexity. Therefore, systems biology approaches are of high value to gain deeper insights. Quantitative modelling requires both, detailed knowledge of kinetic parameters and high computational power. Therefore, such approaches are suitable to model rather small signal transduction modules. Qualitative models provide a better basis for the representation and analysis of large-scale signal transduction networks. Particularly discrete logical modelling is a powerful tool to address important issues, such as detection of network-wide functional interdependencies, identification of intervention targets and predictions on the network dynamics [17,18]. For instance, a literature-based Boolean model has been used to identify cellular myelocytomatosis oncogene (c-Myc) as a putative therapeutic target to treat breast cancer [19]. We previously identified putative therapeutic targets in signal transduction pathways induced by the pathogen *Helicobacter pylori*[20]. With a Boolean model of the DDR, we predicted candidate targets to abolish NF-κB activation, while leaving apoptotic pathways unaffected. The latter targets might be suitable to sensitize carcinomas to DSBs-inducing therapeutics by promoting apoptosis [21]. A literature-based Boolean model of T cell large granular lymphocyte (T-LGL) leukemia has been used to identify potential therapeutic targets and to investigate the dynamics of the signal transduction underlying this disease [22]. Signal transduction emanating from the death receptor has been studied with a discrete logical model. After inactivating certain proteins, the fraction of pathways that lead to a particular cell fate (apoptosis, necrosis or survival) has been determined in dynamical analyses [23].

Here, we present a comprehensive discrete logical model of the response to SSBs and DSBs based on published experimental data. Our dynamical analysis provided new insights into the regulation of p53 and NF-κB in the DDR. We identified candidate target molecules to sensitize tumour cells to DNA damaging therapeutics. By failure mode analysis, we predicted mutations that might contribute to the formation of carcinomas and validated our model with data from published studies.

## Results and discussion

### Logical model of the DDR

**Figure 1:**
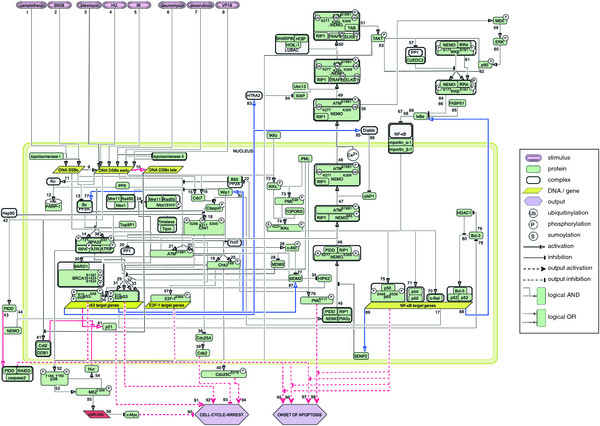
**Logical interaction hypergraph of the DDR.** Genotoxic agents generate DSBs, thereby triggering signal transduction pathways to cell cycle arrest and onset of apoptosis. Arc colours mark earliest (time step 1, black), intermediate (time scale value 2, red) and latest (time scale value 3, blue) interactions, respectively.

Based on quality-controlled literature data, we built a discrete logical model of the response to SSBs and DSBs in human epithelial cells. The model encompasses 96 regulatory components, connected by 98 interactions. It is represented by a logical interaction hypergraph (Figure 1), and a list of logical functions describing the interactions (Additional file 1: Table S1). The numbers assigned to interactions in Figure 1 correspond to the numbers of the logical functions. The network shows the typical structure of signal transduction networks: the input layer is given by stimuli, which damage the DNA, from where signals are being transmitted to and processed in the intermediate layer, finally reaching the output layer (cell cycle arrest and ‘onset of apoptosis’). We chose ‘onset of apoptosis’ instead of ‘apoptosis’ as an output, as this output corresponds to the beginning of apoptotic processes, but not to completion of apoptosis, ie. cell death. The activity levels of most regulatory components (nodes) are represented by Boolean state variables, i.e. they can only attain the values ‘0’ (inactive or absent) or ‘1’ (active or present). Ternary (three-valued) variables were only assigned to phosphorylated ataxia telangiectasia mutated (ATM-P), phosphorylated inhibitor of kappa B kinase (IKK-complex-P) and inhibitor of kappa B α (IκBα). In that way, we took account for the fact that each of those components differs in its functions, depending on whether its activity is low (‘1’) or high (‘2’). Specifically, a low activity of ATM (ATM-P = 1) is required for inactivation of the ATM phosphatase protein phosphatase 2 A (PP2A) [24] (interaction 22). Once PP2A is inactivated, DSBs can induce high activity of ATM (ATM-P = 2), which is now able to phosphorylate further substrates [25] (interaction 23). Similarly, the IKK complex has a low basal activity (IKK-complex-P = 1), which is sufficient for partial degradation of IκBα (decreasing IκBα’s activity level from ‘2’ to ‘1’), leading to activation of proto-oncogene c-Rel (c-Rel) in absence of induced DNA damage [26,27] (interaction 67). Upon induction of DNA damage, the IKK complex attains high activity (IKK-complex-P = 2), which enables more degradation of IκBα (IκBα = 0), enabling the activation of the NF-κB dimers p50-p65-P and p50-p50 [10] (interactions 68, 69). For some structural analyses, we took account for the limited knowledge of time-dependent signal transmission by assigning each interaction to one of three time scale values. Interactions composing the signal transduction pathways leading to activation/inactivation of components that are directly linked to the components “CELL CYCLE ARREST” or “ONSET OF APOPTOSIS” were assigned to time scale value 1, as long as literature data did not indicate a distinct delay. Examples of components that are directly linked to “CELL CYCLE ARREST” or “ONSET OF APOPTOSIS” are the transcription factors. Time scale value 2 was assigned to interactions that also lead to cell cycle arrest, apoptosis, or anti-apoptosis, but were shown to occur distinctively later than interactions of time scale value 1. For example, p53-induced protein with a death domain (PIDD) binds to NEMO (interaction 44, time scale 1), and later, PIDD binds to RIP1-associated ICH-1/CED-3 homologous protein with a death domain (RAIDD) (interaction 43, time scale 2) [28]. Time scale value 2 was also assigned to interactions linked directly to the regulatory components “CELL CYCLE ARREST” or “ONSET OF APOPTOSIS”. Activation of proteins that initiate switching off parts of the DDR was assigned time scale value 3. This was based on the assumption that these events occur during the latest phase of the DDR. For instance, Wip1 interrupts signal transduction pathways by dephosphorylating ATM and other proteins [29] (e.g. interaction 21). Accordingly, induction of Wip1 expression [30] (interaction 82) has been assigned to time scale value 3. Detailed information on assignments of time scale values are given in Additional file 1: Table S1. For most analyses, we simulated the DDR at time scale value 2, i.e., at a time before feedback inhibition comes into play. Our study focused on inhibitions and molecular defects interfering with cell cycle arrest, apoptosis, or anti-apoptosis. As follows from the considerations above, only time scale value 2 pertains to maximum activity of all components promoting cell cycle arrest, apoptosis, or anti-apoptosis. Hence, for this time scale value, the sensitivity of the simulation results to changes in time scales of interactions should be minimal. 

For dynamical analyses, we took account for the knowledge of time-dependent signal transmission by defining three priority classes (see below).

### Validation of the predictive quality of the model

In order to validate the predictive quality of our model, we evaluated simulations on the basis of published studies on epithelial cells (Table 1). We inactivated certain proteins in the model and then calculated the logical steady state of the model at time scale value ‘2’, i.e. prior to onset of negative Feedback inhibition. Cells can be sensitized to DNA damaging therapy by events that promote cell death [31]. Blockage of cell cycle arrest can cause mitotic catastrophy, a form of cell death [13], whereas blocking of the anti-apoptotic transcription factor NF-κB promotes apoptosis [14]. 

**Table 1:** Validation of the model on reported effects with functional p53

**Inactivated Protein**	**DNA damage**	**Reported effects with functional p53**	**References**	**Model prediction**
ATM	DSBs	· cell cycle arrest diminished	Lavin 2008	· cell cycle arrest blocked
			Bolderson et al. 2009	· anti-apoptotic NF-κB (p50-p65-P) blocked
		· cell death enhanced		
				· apoptosis diminished
			Tofilon and Camphausen 2009	
ATR	SSBs	· cell death enhanced	Flatten et al. 2005	· cell cycle arrest diminished
			Wagner and Kaufmann 2010	
Chk1	SSBs	· cell cycle arrest diminished	Tse et al. 2007	· Cdc25A degradation diminished (degradation of Cdc25A leads to cell cycle arrest)
			Ma et al. 2011	
		· cell death enhanced		· p53 activation (causes apoptosis or cell cycle arrest) diminished
			Garret and Collins 2011	
Chk2	DSBs	cell death reduced	Morgan et al. 2010	· cell cycle arrest diminished
			Ma et al. 2011	
				· apoptosis diminished
			Garret and Collins 2011	· p53 activation (causes apoptosis or cell cycle arrest) diminished
TAK1	SSBs	· cell death enhanced	Martin et al. 2011	· cell cycle arrest diminished

Inactivation of ATM blocked all pro-survival pathways (cell cycle arrest and NF-κB activation) in the response to DSBs. This is confirmed by studies in which ATM inhibition sensitizes cells to agents causing DSBs [32-34].

Ataxia telangiectasia and rad3-related protein (ATR) inactivation blocked two pathways leading to cell cycle arrest (downregulation of c-Myc, expression of p21) in response to SSBs in our model. This is in agreement with the reported potentiation of SSBs-induced cell death by ATR inactivation in carcinoma cells [35,36].

In our simulation of the response to SSBs, loss of checkpoint kinase 1 (Chk1) blocked one of two pathways promoting cell division cycle 25 A (Cdc25A) degradation. Degradation of Cdc25A leads to cell cycle arrest. Additionally blocked was one pathway leading to activation of p53, a pro-apoptotic and cell cycle arresting protein. Thus, loss of Chk1 suppressed pathways leading to cell cycle arrest and apoptosis. Hence, our results do not indicate, whether Chk1 inhibition sensitizes cells to SSBs inducers. Chk1 inhibition was demonstrated to increase the cytotoxicity to topoisomerase I (TOPI) inhibitors by diminishing cell cycle arrest in carcinoma cells with functional p53 [37-39]. As previously proposed, a partial suppression of p53 activation diminishes predominantly its apoptotic function and to a lesser extent its cell cycle arresting function [15]. This effect might contribute to the sensitization by Chk1 inhibition, but is not captured by the model.

In response to ionizing radiation, absence of Chk2 in our model blocked cell cycle arresting phosphorylation of Cdc25C, and one of two pathways leading to degradation of Cdc25A. On the other hand, activation of the pro-apoptotic effectors promyelocytic leukemia (phosphorylated at serine 117) (PML-PS117) and phosphorylated adenovirus E2 gene promoter region binding factor 1 (E2F-1-P), and one p53 activating pathway are blocked. Hence, the numbers of both, cell cycle arresting and apoptotic pathways were reduced. The simulation did not indicate, whether Chk2 inhibition confers sensitization or protection from cell death caused by ionizing radiation. In most studies, Chk2 inhibition diminished cell death caused by ionizing radiation [37,39]. Correspondingly, Chk2 knockdown protects MIA PaCa-2 carcinoma cells against ionizing radiation [40].

When simulating the response to camptothecin (inhibitor of TOPI) in the model, inhibition of TGF-β activated kinase 1 (TAK1) abolished two cell cycle arresting pathways (c-Myc downregulation and p21-mediated cell cycle arrest). Hence, the model indicates a sensitizing effect of TAK1 knockdown, which was demonstrated in carcinoma cell lines treated with camptothecin [41].

Moreover, putative therapeutic targets for the sensitization of tumours with dysfunctional p53 have been proposed (Table 2). We compared the response to the topoisomerase II (TOPII) inhibitor doxorubicin in absence of p53 only with the response in absence of p53 and ATM. In the absence of only p53, four cell survival pathways were still active, i.e. activation of anti-apoptotic NF-κB, cell cycle arrest induced by c-Myc downregulation, Cyclin-dependent kinase 2 (Cdk2) inhibition, and phosphorylation of Cdc25C. When p53 and ATM were absent, no cell survival pathway was activated by doxorubicin in the model. Accordingly, the ATM inhibitor KU-55933 sensitizes p53-deficient human carcinoma cells to doxorubicin. Moreover, p53-deficient breast and lung tumours showed higher sensitivity to genotoxic chemotherapy when ATM is inactive as well [16]. 

**Table 2:** Validation of the model on reported effects without functional p53

**Inactivated Protein**	**DNA damage**	**Reported effects without functional p53**	**References**	**Model prediction**
ATM	DSBs	· cell death enhanced	Jiang et al. 2009	· cell cycle arrest blocked
				· anti-apoptotic NF-κB (p50-p65-P) blocked
				· apoptosis diminished
Chk1	SSBs	· cell cycle arrest diminished	Tse et al. 2007	· Cdc25A degradation diminished (degradation of Cdc25A leads to cell cycle arrest)
			Ma et al. 2011	
		· cell death enhanced	Garret and Collins 2011	
		· these effects of Chk1 inhibition are		
		more pronounced in p53-deficient cells than in cells with a functional p53		
Chk2	DSBs	· cell death enhanced or cell death not enhanced?	Jiang et al. 2009	· cell cycle arrest diminished
				· apoptosis diminished
			Anderson et al. 2011	

In the p53-deficient model, TOPI inhibitors still induced cell cycle arrest. Additional loss of Chk1 abolished one of the pathways leading to degradation of Cdc25A, a phosphatase necessary for cell cycle progression. Apoptotic pathways in p53-deficient cells were not suppressed by inactivation of Chk1. Hence, our model indicated that p53-deficient cells might be sensitized to SSBs inducers by inhibition of Chk1. Indeed, the aforementioned sensitization to TOPI inhibitors by Chk1 inhibition was reported to be more pronounced when p53 is dysfunctional [37-39]. Accordingly, preclinical studies support the combination of Chk1 inhibitors with SSBs inducers particularly for treatment of p53-deficient tumours [37,39].

In the model, inactivation of Chk2 in absence of p53 reduced the number of cell cycle arresting and pro-apoptotic pathways. The sensitivity of tumours with dysfunctional p53 to DSB-causing agents was reported to be potentiated by inactivation of Chk2 [16]. In contrast, another study showed no pronounced potentiation of cell death by Chk2 inhibition in carcinoma cells with a loss of function mutation in p53 [42]. As suggested by our simulations, whether Chk2 inhibition potentiates cell death caused by DSBs might depend on the genetic background, providing a possible explanation for the conflicting experimental data.

In summary, our simulations recapitulated most published studies on the sensitivity of carcinoma cells to DNA damaging agents after inactivation of a certain protein. These results support the suitability of the model for the generation of predictions.

### Network-wide interdependencies

**Figure 2:**
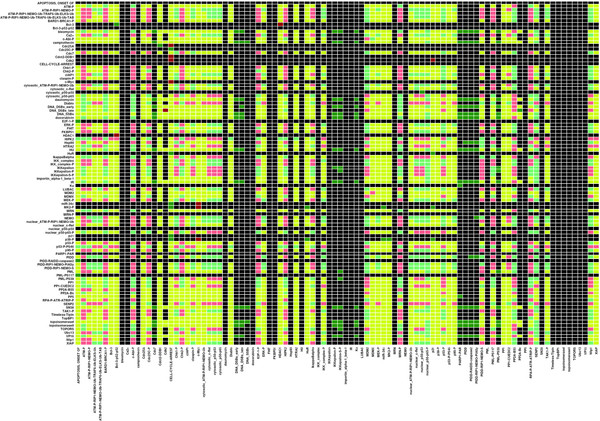
**Dependency matrix.** Interdependencies between all pairs of regulatory components in the model are displayed. The colour of a matrix element M*ij* defines the type of impact of a component *i* (left hand side) on a component *j* (bottom). Colour codes: dark green, strong activator; turquois green, weak activator; dark red, strong inhibitor; pink, weak inhibitor; yellow, ambivalent factor; black, no effect. ‘-P’ = phosphorylation, ‘-S’ = sumoylation, ‘-Ub’ = ubiquitinylation.

Network-wide causal relationships between all pairs of regulatory components are displayed in the dependency matrix (Figure 2). Two components have a causal relationship, if a sequence of adjacent components, a pathway, links them. As the large fraction of yellow matrix elements in Figure 2 illustrates, in most causal relationships between two components *i* and *ji* is an ambivalent factor for *j*. In other words, *i* has an activating as well as an inhibiting influence on another component *j*. Usually, the activating influence becomes operational at another time scale than the inhibiting influence. ATM for instance phosphorylates, i.e. has an activating influence on Chk2 [43] (interaction 25). However, ATM phosphorylates p53 as well [44,45] (interaction 31), leading to expression of Wip1 later (at time scale value 3) [30] (interaction 82). Wip1 in turn deactivates Chk2 by means of dephosphorylation [46] (interaction 25). Therefore, the activation of Chk2 by ATM is counteracted by the ATM-dependent deactivation of Chk2 by Wip1. Thus, ATM is an ambivalent factor for Chk2, as the yellow matrix element in Figure 2 indicates. As the high frequency of coincidences of activating and inhibiting relationships indicates, most pathways become inactivated in a later phase of the DDR. Moreover, these coincidences suggest an important role of crosstalk in the DDR. 

### Dynamics of the DDR

**Figure 3:**
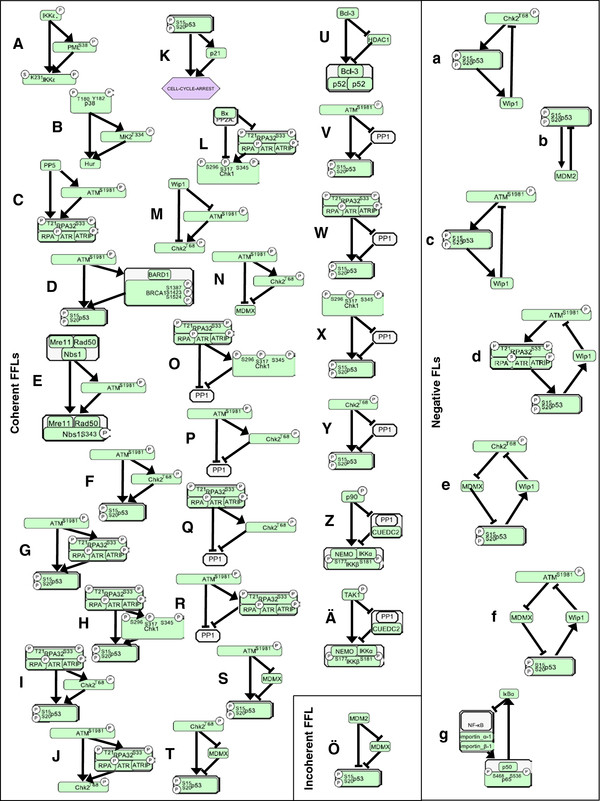
**Feed-forward loops (FFLs) and Feedback loops (FLs) in the logical model.** Coherent FFLs of type 1 with AND logic (A-E), or OR logic (F-K), respectively; coherent FFLs of type 2 with AND logic (L, M); coherent FFLs of type 3 (N-R); coherent FFLs of type 4 (S-Ä), and an incoherent FFL of type 2 (Ö). Among negative FLs (a-g), only the functional (in the logical model) are shown. Letters in circles: P = phosphorylation, S = sumoylation.

Feed-forward loops (FFLs) and Feedback loops (FLs) can play decisive roles in the processing of the signals, which are being transmitted in signal transduction networks. Moreover, they may profoundly influence the dynamics (temporal behaviour) of a signal transduction network [47]. For these reasons, we identified FFLs (Figure 3). They appear in two groups, those with ‘AND gates’ and those with ‘OR gates’. For example, ‘AND gated’ is the activation of sumoylated and phosphorylated IKKϵ (IKKϵ-S-P) by IKKϵ-P and PML-P (Figure 3A), as IKKϵ-S-P activation requires both proteins, i.e. IKKϵ-P AND PML-P. ‘OR gated’ is for instance the activation of p53-P by either ATM-P or Chk2-P (Figure 3F), as either ATM-P OR Chk2-P phosphorylates p53. 

Coherent FFLs of type 1 with ‘AND gates’ may delay the transmission of activating signals [48]. Such FFLs in the model are shown in Figure 3A-E. Coherent FFLs of type 4 can have the same function [48]; they are shown in Figure 3S-Ä.

As also reported by Mangan and Alon [48], transmission of the fade away of signals (‘OFF’ signals) in a pathway could be delayed by coherent type 1 FFLs with ‘OR gate’ (Figure 3F-K), by coherent type 2 FFLs with ‘AND’ gate (Figure 3L and M), as well as by the coherent type 3 FFLs (Figure 3N-R).

Incoherent type 2 FFLs with ‘AND gate’ may accelerate the transmission of ‘OFF’ signals [48]. We found only one example (Figure 3Ö).

In summary, all but one (Figure 3Ö) FFLs identified may delay either ‘ON’ or ‘OFF’ signals, thereby transmitting only long-term signals. Moreover, we found that most of these FFLs include either p53, or its regulators. Taken together, short-term signals arising from noise rather than from DNA damage might be filtered out. The same regards signals arising from minor damage of DNA, which becomes rapidly repaired. Only long-term signals from more severe DNA damage would be transmitted to and activate p53. Such a careful regulation seems reasonable in light of the well-known key role of p53 in determining cell fate (survival or apoptosis) after DNA damage. Indeed, such a regulation of the actelyation of p53 involving so far unknown FFLs has been proposed [49]; our results provide evidence for a regulation of p53 phosphorylation by only long term signals and provide candidate FFLs for that mechanism. As we found moreover, the FFL in Figure 3A might delay ‘ON’ signals transmitted to IKKϵ-S-P. Similarly, the FFLs in Figure 3Z and Ä could delay ‘ON’ signal transmission to the IKK complex. In both cases, short-term signals could be filtered out. IKKϵ-S-P and the IKK complex mediate activation of NF-κB. Similarly to the mentioned control of p53, such a careful regulation of NF-κB seems reasonable in light of its mayor role in counteracting apoptosis.

Next, we identified FLs that are functional in the logical model (Figure 3a-g). All of them are negative. The presence of a negative FL is necessary for stable oscillations [50]. Again, most FLs (Figure 3a-f) contain p53, whereas the FL in Figure 3g contains the NF-κB dimer p50-p65. In the latter FL, NF-κB drives the expression of its own inhibitor IκBα. This FL was shown to cause oscillatory behaviour of NF-κB in a multitude of cells and treatment conditions [51]. Also the FLs in Figure 3a-c have been studied previously with ordinary differential equation or stochastic models as well as experimentally in cells exposed to ionizing radiation [8]. In a logical approach, effects of varied (i) degradation rates of MDM2, (ii) transcriptional activities of p53, and (iii) DNA damage levels on the dynamic behaviour of the MDM2-p53 circuit has been studied. It has been shown that variations in parameter values (e.g. corresponding to mutations) can lead to only four different scenarios of dynamical behaviour of the network [52]. Recently, the feedback-controlled oscillations of p53 were proposed to impact the ultimate cell fate decision [9]. As our results suggest, the negative FLs in Figure 3d-f might cause oscillations of p53 levels *in vivo* as well.

**Figure 4:**
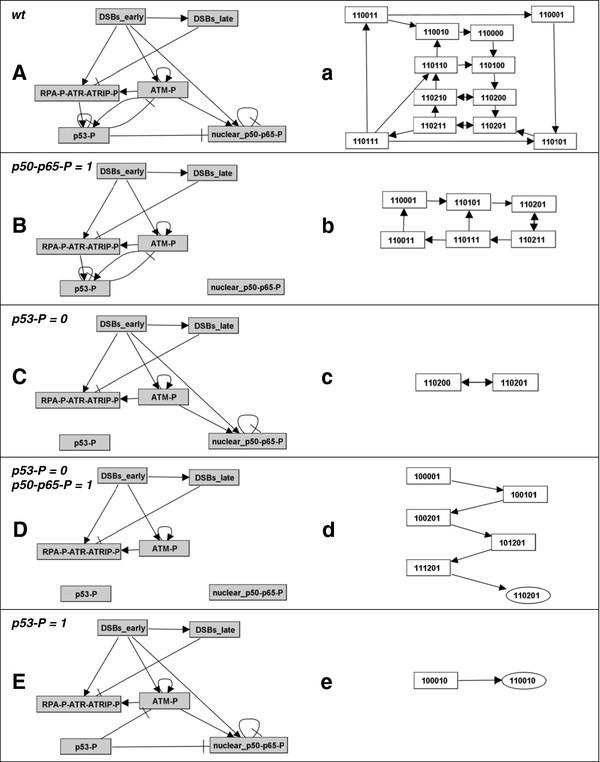
**Dependence of the network dynamics on p53 and NF-κB**. A-E: Interaction graphs of the core network comprising only the regulatory components ‘DSBs early‘, DSBs late‘, ‘RPA-P-ATR-ATRIP-P‘, ‘ATM-P‘, p53-P‘, and ‘nuclear p50-p65-P‘. Shown are the networks for the wildtype **(A)**, the mutant with constitutively active p50-p65-P **(B)**, p53-deficiency **(C)**, both constitutively active p50-p65-P and p53-deficiency **(D)**, and constitutively active p53 **(E)**. a-e: corresponding state transition graphs, which illustrate the dynamical behaviour of the networks. The nodes of the state transition graphs gives the activity levels of ‘DSBs early‘ (first digit), ‘DSBs late‘ (second digit), ‘RPA-P-ATR-ATRIP-P‘,(third digit), ‘ATM-P‘ (fourth digit), p53-P‘ (fifth digit), and ‘nuclear p50-p65-P‘ (sixth digit). State transition graphs a-c show only attractors. In d and e, complete state transition graphs are given, wherein the logical steady states are marked with ellipses.

In order to study the terminal fate (attractors) of the network, we reduced it to a model with conserved attractors. Previously, a method has been proposed to reduce Boolean models to their functional interactions. However, this method is only applicable to models of intermediate dimension (i.e. maximal 20 variables) [53]. Therefore, we used a different network reduction technique, which is applicable to large-scale models (see Methods). The reduced model contains only the regulatory components DSBs early, DSBs late, RPA-ATR-ATRIP-P, ATM-P, p53-P and NF-κB (nuclear p50-p65-P) (Figure 4). We calculated the state transition graph of the reduced model by using an asynchronous updating schedule with three priority classes. The state transitions that were assigned to priority classes 1, 2, and 3 coincide with the interactions of time scale values 1, 2, and 3, respectively. Hence, state transitions involving activations of RPA-ATR-ATRIP-P, ATM-P, p53-P or nuclear NF-κB were assigned to priority class 1; priority class 2 embraces the subsequent state transitions leading to activation of ‘DSBs late’ by ‘DSBs early’. State transitions coinciding with the initiation of the inactivation of signal transduction pathways, i.e., the downregulation of RPA-ATR-ATRIP-P, ATM-P, p53-P and NF-κB, constitute priority class 3. 

We emphasize that the attractors of the model variants correspond to the fate of the DDR before the cell either completes DNA repair or dies. In response to DSBs, the model (Figure 4A) finally enters a complex cyclic attractor (Figure 4a). This suggests the cellular network might transit through an intertwined cycle of states before completion of either DNA repair or apoptosis. Negative feedbacks are necessary for cyclic attractors [50]. We thus aimed to elucidate in more detail the roles of the identified feedbacks (Figure 3) in generating the cyclic attractor. For this purpose, we calculated state transition graphs for model variants with interrupted feedbacks. Models with constitutively active NF-κB (Figure 4B) or deficiency of p53-P (Figure 4C) still enter cyclic attractors (Figure 4b and c). Similarly, the model variant with deficiency of NF-κB enters a cyclic attractor as well (not shown). In contrast, the model variant with both p53-deficiency and constitutively active NF-κB (Figure 4D) enters a logical steady state (marked with an ellipse in Figure 4d). Even constitutive activation of only p53-P is sufficient to direct the network into a logical steady state (Figure 4e). The network reduction we applied can lead to loss of trajectories in the STG. Therefore, not every trajectory in the STG of the full model might have a counterpart in the STG of the reduced model [54]. Consequently, the reduced model variant’s attractors we identified might be different from those of the full model variants (i.e. of the model variants before the reduction was done). Therefore, we checked for each of the five reduced model variant’s attractors (Figure 4), whether it is equivalent to the attractor of the corresponding full model variant. In general, any attractor is either a logical steady state or a cyclic (simple loop or complex loop) attractor [50,55]. Whereas we were able to identify the logical steady states of the full model variants, their state spaces are too big to identify cyclic attractors. Therefore, if a full model variant has no logical steady state, we inferred the presence of a cyclic attractor. The identified logical steady states are independent of the updating scheme applied [18], and therefore, insensitive to changes in the priority classes. As our aim now was only to check for the type of attractor (steady state or cyclic attractor), there was no need to specify priority classes. As we found, neither the wildtype full model, nor its variant with constitutively active NF-κB or deficiency of p53-P enter a logical steady state (Additional file 1: Table S2, columns A-C). Hence, these full model variants should enter cyclic attractors; the results are in agreement with the identified cyclic attractors of the corresponding reduced model variants (Figure 4a-c). The full model variants with both p53-deficiency and constitutively active NF-κB (Additional file 1: Table S2, column D) as well as the full model variant with constitutively activative p53-P (Additional file 1: Table S2, column E) enter logical steady states. Again, the results agree with the results from the analyses of the reduced model counterparts (Figure 4d and e). In addition, none of the full model variants contains a functional positive FL (data not shown); thus, this necessary condition for bi- or multistability [50] is not fulfilled. Therefore, each full model variant possesses only a single attractor. Again, our results coincide with the finding that each reduced model variant possesses only a single attractor.

We conclude that all attractors of the reduced model variants correspond to those of the full model variants. Both, the results gained from the analyses of the attractors and the identified functional FLs independently suggest an essential role of p53 and NF-κB in the generation of cyclic attractors of the DDR. This and the prevalence of p53, and NF-κB in the FFLs support the importance of these proteins in governing the dynamics of the DDR.

### Candidate target proteins for sensitization of carcinomas to therapies

To identify putative targets for sensitization of carcinomas to therapy, we simulated treatments with agents causing SSBs (inhibitors of TOPI) or only DSBs (e.g. inhibitors of TOPII). p53, homeo-domain interacting protein kinase 2 (HIPK2), ATM or Chk2 are frequently mutated and inactive in carcinoma cells [56-59], therefore, we simulated treatment with inhibitors of TOPI or TOPII in the absence of these proteins. In order to simulate the behaviour of the network before the onset of feedback inhibition, we chose the time scale value ‘2’ of the model. We calculated minimal intervention sets of targets, whose inhibition might sensitize tumours by fulfilling three intervention goals: (i) blocking cell cycle arrest, (ii) blocking activation of anti-apoptotic NF-κB, and (iii) keeping at least one pathway activating ‘onset of apoptosis’ intact. In presence of (therapeutically induced) severe DNA damage inhibitors that fill out goal (i) would eliminate tumour cells by mitotic catastrophy, and inhibitors fulfilling goals (ii) and (iii) would potentiate apoptosis. We identified 85 sets of molecular targets that could sensitize tumour cells to therapies inducing SSBs or DSBs (Table 3), and protein sets containing putatively less suitable targets (Additional file 1: Table S3). ATM deficiency in the model already fulfils the intervention goals in presence of DSBs. Hence, we found no sensitization target for such conditions. This result agrees with most studies, showing that ATM inhibition sensitizes cells to therapeutics causing DSBs [32]. Accordingly, cells isolated from Ataxia telangiectasia (dysfunctional ATM) patients show enhanced radiosensitivity [33]. For certain sets, inhibitions of the target proteins might specifically sensitize tumour cells with the indicated mutation, but allow normal cells to survive by entering cell cycle arrest (asterisks in Table 3). 

**Table 3:** Candidate targets to sensitize epithelial tumours to therapy

**Mutated Protein in Tumor**	** Candidate targets to sensitize tumours to therapies inducing**	
	**DNA SSBs**	**DNA DSBs**
**p53**	ATM-P & ATR	ATM-P
	ATM-P & Chk1-P & Chk2-P	ATR & Chk2-P & FKBP51*
	ATM-P & Chk2-P & c-Rel	ATR & Chk2-P & IKK_complex-P*
	ATM-P & Chk2-P & IKK_complex-P	ATR & Chk2-P & IKKϵ-P*
	ATR & Chk2-P & FKBP51*	ATR & Chk2-P & p50-p65-P*
	ATR & Chk2-P & IKK_complex-P*	Chk2-P & IKK_complex-P & LUBAC*
	ATR & Chk2-P & IKKϵ-P*	Chk2-P & IKK_complex-P & MK2-P*
	ATR & Chk2-P & p50-p65-P*	Chk2-P & IKK_complex-P & TAK1-P*
	ATR & MRN	Chk2-P & IKK_complex-P & TRAF6*
	Chk1-P & Chk2-P & MRN	Chk2-P & IKK_complex-P & XIAP*
	Chk2-P & c-Rel & MRN	MRN
	Chk2-P & IKK_complex-P & LUBAC*	PP5
	Chk2-P & IKK_complex-P & MK2-P*	
	Chk2-P & IKK_complex-P & MRN	
	Chk2-P & IKK_complex-P & TAK1-P*	
	Chk2-P & IKK_complex-P & TRAF6*	
	Chk2-P & IKK_complex-P & XIAP*	
	PP5	
**HIPK2**	ATM-P & ATR	ATM-P
	ATM-P & Chk1-P & Chk2-P	MRN
	ATM-P & Chk2-P & c-Rel	PP5
	ATM-P & Chk2-P & IKK_complex-P	
	ATR & MRN	
	Chk1-P & Chk2-P & MRN	
	Chk2-P & c-Rel & MRN	
	Chk2-P & IKK_complex-P & MRN	
	PP5	
**ATM**	ATR*	No targets found.
	Chk1-P & Chk2-P*	
	Chk2-P & c-Rel*	
	Chk2-P & IKK_complex-P*	
	PP5	
**Chk2**	ATM-P & ATR	ATM-P
	ATM-P & Chk1-P*	ATR & BARD1-BRCA1-P & FKBP51*
	ATM-P & c-Rel*	ATR & BARD1-BRCA1-P & IKK_complex-P*
	ATM-P & IKK_complex-P*	ATR & BARD1-BRCA1-P & IKKϵ-P*
	ATR & BARD1-BRCA1-P & FKBP51*	ATR & BARD1-BRCA1-P & p50-p65-P *
	ATR & BARD1-BRCA1-P & IKK_complex-P*	ATR & FKBP51 & p53-P*
	ATR & BARD1-BRCA1-P & IKKϵ-P*	ATR & IKK_complex-P & p53-P*
	ATR & BARD1-BRCA1-P & p50-p65-P *	ATR & IKKϵ-P & p53-P*
	ATR & FKBP51 & p53-P*	ATR & p53-P & p50-p65-P*
	ATR & IKK_complex-P & p53-P*	IKK_complex-P & LUBAC & p53-P*
	ATR & IKKϵ-P & p53-P*	IKK_complex-P & MK2-P & p53-P*
	ATR & MRN	IKK_complex-P & p53-P & TAK1-P*
	ATR & p50-p65-P & p53-P*	IKK_complex-P & p53-P & TRAF6*
	Chk1-P & MRN*	IKK_complex-P & p53-P & XIAP*
	c-Rel & MRN*	MRN
	IKK_complex-P & LUBAC & p53-P*	PP5
	IKK_complex-P & MK2-P & p53-P*	
	IKK_complex-P & MRN*	
	IKK_complex-P & p53-P & TAK1-P*	
	IKK_complex-P & p53-P & TRAF6*	
	IKK_complex-P & p53-P & XIAP*	
	PP5	

^*^ Sets of targets whose inhibition might specifically sensitize tumor cells with the indicated mutation, but allow normal cells to survive by entering cell cycle arrest.

Some predicted target sets include ATR or Chk1, which beside their contributions to the DDR are essential for proliferation. Nevertheless, partial and transient inhibition of ATR or Chk1 during DNA damage diminishes cell cycle arrest rather than proliferation [35,37]. Additionally, some protein target sets that sensitize Chk2-deficient tumours include p53. Although p53 can promote apoptosis, it mediates predominantly cell cycle arrest in Chk2-deficient tumours, resulting in tumour cell survival [15]. Correspondingly, p53 inhibition might sensitize certain tumours to therapeutic treatment [60]. Hence, inhibition of p53 in Chk2-deficient cells seems reasonable. Taken together, we predict putative protein target sets that might sensitize tumours carrying certain mutations to therapeutic interventions. Our candidate target sets in Table 3 include all published sensitization targets in Tables 1 and 2. However, with the exception of ATM, inhibiting the published sensitization targets in Tables 1 and 2, blocks only part of the cell survival pathways of the model in tumours containing certain mutations. In contrast, our proposed target sets (Table 3) might block all cell survival pathways of the model in tumours containing certain mutations. Hence, our candidate targets might sensitize tumours to DNA damaging therapeutics with higher efficiency.

### Simulation of genetic disorders

Next, we aimed to enlighten the DDR in genetic diseases. For this purpose we inactivated in our model the protein whose defect causes a given disease. Then, we simulated the response to SSBs and DSBs simultaneously at time scale value ‘2’, and evaluated our *in silico* results based on published data. For investigations of the feedback control of the DDR, we simulated at time scale value ‘3’ (Table 4). The disease Ataxia telangiectasia (ATM-deficiency) has been associated with defects in the activation of p53, G1/S, intra-S, and G2/S cell cycle checkpoints, genomic instability, enhanced radiosensitivity and increased incidence of lymphoid tumours [33,61]. In our simulation, loss of ATM blocked p53 activation and p21 expression, resulting in abolished cell cycle arrest by these proteins. Additionally, the cell cycle promoting protein c-Myc became expressed, and abolished another cell cycle arrest pathway. Cell cycle checkpoint defects are known to contribute to genomic instability, which promotes tumorigenesis [11], and increased cell death by mitotic catastrophy [13]. The abolished activation of NF-κB in the model might further promote apoptosis, although p53-dependent apoptosis was blocked as well. Moreover, in absence of ATM we identified in our model the loss of many signalling pathways involved in the regulation of p53 and NF-κB target genes. 

**Table 4:** Simulation of genetic disorders

**Genetic disorder**	**Molecular cause**	**Reported defects**	**References**	**Model prediction**
Ataxia Telangiectasia	ATM dysfunctional	molecular defects	Jorgensen & Shiloh 1996	· p53 activation blocked
				· anti-apoptotic NF-κB (p50-p65-P) blocked
		· p53 activation blocked		
				· cell cycle arrest diminished
				· some feedback loops, which terminate signal transduction pathways blocked
			Lavin 2008	
		cellular defects		
		· cell cycle checkpoints all defect		
		· genomic instability		
		· radiosensitivity enhanced		
		clinical features		
		· incidence of lymphomas increased		
Ataxia Telangiectasia-Like-Disorder	Mre11 dysfunctional	cellular defects	Lavin 2008	· cell cycle arrest diminished
		· cell cycle arrest diminished		· some feedback loops, which terminate signal transduction pathways blocked
		· genomic instability		
		· radiosensitivity enhanced		
Nijmegen Breakage Syndrome	Nbs1 dysfunctional	molecular defects	Antoccia et al. 2006	· activation of ATR by ATM abolished
		· ATR activity reduced		
				· p21 expression blocked
				· cell cycle arrest diminished
			Lavin 2008	· no BRCA1 phosphorylation
		· p21 expression reduced		· some feedback loops, which terminate signal transduction pathways blocked
		cellular defects		
		· cell cycle checkpoints all defect		
		· sensitivity to agents causing DSBs enhanced		
		· DNA repair diminished		
		clinical features		
		· incidence of lymphomas increased		
Nijmegen Breakage Syndrome-Like Disorder	Rad50 dysfunctional	cellular defects	Barbi et al. 1991	· cell cycle arrest diminished
		· cell cycle arrest diminished		· some feedback loops, which terminate signal transduction pathways blocked
			Waltes et al. 2009	
		· genomic instability		
		· radiosensitivity enhanced		
ATR-Seckel Syndrome	ATR level reduced	molecular defects	O’Driscoll et al. 2004	· activation of p53 and Chk1 by ATR diminished
		· phosphorylations of Chk1 and p53 by ATR reduced		
				· cell cycle arrest diminished

Ataxia telagiectasia-like-disorder (Mre11-deficiency) is also associated with defective induction of cell cycle arrest, genomic instability, and enhanced radiosensitivity [33]. As Mre11 in the model is a subunit of the MRN complex, which solely activates ATM, the blocked pathways are the same as in the Ataxia telangiectasia simulation. The same is true for Nijmegen breakage syndrome (Nbs1-deficiency), as in the model also Nbs1 is only a MRN complex subunit. Nijmegen breakage syndrome has moreover been reported to diminish DNA repair [33,62]. However, DNA damage induced cell cycle arrest promotes DNA repair [11]. Hence, the abolishment of cell cycle arrest by p53 phosphorylation, p21 expression, and c-Myc downregulation in the simulation might contribute to lost repair capabilities. Moreover, breast cancer 1, early onset (BRCA1) is essential for homologous recombination repair of DSBs [1]. ATM-dependent phosphorylation of BRCA1 is averted by loss of Nbs1 in the model, possibly further contributing to DNA repair deficiencies.

Also Rad50 in the model is a subunit of the MRN complex, which solely activates ATM. Hence, the pathways blocked in Nijmegen breakage syndrome-like disorder (Rad50-deficiency) [63,64] are identical to those in the Ataxia telangiectasia simulation.

A reduction of the ATR level causes ATR-Seckel syndrome. Hence, ATR-dependent phosphorylations of Chk1 and p53 are diminished, whereas ATM-dependent responses to ionizing radiation remain intact. There is no pronounced radiosensitivity, and no enhanced incidence of cancer [65]. In line with this report, ATR inactivation in the model did not affect ATM-dependent signalling induced by ionizing radiation. Instead, ATR-deficiency abolished cell cycle arrest mediated by p21 expression and c-Myc downregulation in the model. However, these cell cycle arresting pathways could still be active in presence of an ATR level as low as in ATR-Seckel syndrome cells.

### Molecular defects putatively contributing to carcinogenesis

DDR defects that diminish apoptosis and cell cycle arrest are well known to contribute to carcinogenesis by promoting uncontrolled proliferation [11]. We aimed to identify putatively relevant malfunctions in the DDR in epithelial cells. For this purpose, we simulated the response to both SSBs and DSBs simultaneously. Again, we chose the time scale value ‘2’ of the model. By calculating minimal intervention sets, we identified protein defects (i.e. constitutive activations and inactivations that in combination block both, ‘onset of apoptosis’ and cell cycle arrest in the model. *In vivo*, constitutive activation of a protein can result from constitutive expression or gain of function mutations of the gene encoding that protein, whereas constitutive inactivation can result from loss of function mutations. Alternatively, such defects of a protein might result from mutations of a gene encoding another protein that regulates the relevant protein. We found 117 combinations of defects that putatively contribute to carcinogenesis by allowing cells with damaged DNA to proliferate (Table 5). From our search, we excluded activations and inactivations that according to literature data might counteract uncontrolled proliferation (Additional file 1: Table S4). By doing so, we accounted for protein functions being relevant to carcinogenesis, but are not captured by the model. Each row gives an alternative combination of relevant defects (Table 5). Here, [0] means inactive; [1]: constitutive active at level ‘1’; [2]: constitutively active at level ‘2’. Among the results are inactivities of the known or suspected tumour suppressors ATM, the MRN complex subunit Nbs1, fragile histidin triad gene (FHIT), p53, BRCA1, as well as Chk2, and activities of the known oncogene MDM2 [66,67]. In 21 combinations, the NF-κB dimer p50-p65 was found to be constitutively active, as frequently observed in tumours [68]. Carcinogenesis might also be promoted by constitutive active IκBα or the IKK complex (e.g. sets 2, 13). Alternatively, constitutive active IκBα or the IKK complex may promote carcinogenesis in combination with other molecules (e.g. sets 110, 6). To our knowledge, most of the putative carcinogenic mutations we found have not been implicated in tumorigenesis previously. This gap of knowledge could be explained by ‘low penetrance genes’ assumed to confer cancer susceptibility or resistance [66,69,70]. These were proposed to act in combination in a dosage-dependent manner to incrementally determine cancer predisposition. A major obstacle to their identification is the vast number of possible combinations of mutations to be tackled experimentally [66,71]. However, our results may provide a valuable preselection for follow-up experiments. 

**Table 5:** Combinations of constitutive activations and inactivations putatively leading to carcinogenesis

**No.**	**Events putatively contributing to carcinogenesis**		
1	[0]PP5	& [0]PIDD-RAIDD-caspase2	
2	[0]PP5	& [0]IkappaBalpha	
3	[0]PP5	& [0]PIDD	
4	[0]PP5	& [0]Hsp90	
5	[0]PP5	& [1]p90-P	
6	[0]PP5	& [2]IKK_complex-P	
7	[0]PP5	& [1]MEK-P	
8	[0]PP5	& [1]ERK-P	
9	[0]PP5	& [1]cytosolic_p50-p65	
10	[0]PP5	& [1]nuclear_p50-p65-P	
11	[0]PP5	& [1]TAK1-P	
12	[1]Wip1	& [0]Hsp90	
13	[1]Wip1	& [0]IKK_complex-P	& [0]PIDD-RAIDD-caspase2
14	[1]Wip1	& [0]PIDD	& [0]IKK_complex-P
15	[1]Wip1	& [0]IKK_complex-P	& [1]cytosolic_p50-p65
16	[1]Wip1	& [0]IKK_complex-P	& [1]nuclear_p50-p65-P
17	[0]ATM-P	& [0]Hsp90	& [0]Chk2-P
18	[0]ATM-P	& [1]PP2A-Bx	& [0]PIDD-RAIDD-caspase2
19	[0]ATM-P	& [1]PP2A-Bx	& [0]IkappaBalpha
20	[0]PIDD	& [0]ATM-P	& [1]PP2A-Bx
21	[0]FHIT	& [0]ATM-P	& [0]Hsp90
22	[0]ATM-P	& [0]Hsp90	& [1]PP2A-Bx
23	[1]p90-P	& [0]ATM-P	& [1]PP2A-Bx
24	[0]ATM-P	& [1]PP2A-Bx	& [2]IKK_complex-P
25	[0]ATM-P	& [1]MEK-P	& [1]PP2A-Bx
26	[0]ATM-P	& [1]ERK-P	& [1]PP2A-Bx
27	[0]ATM-P	& [1]cytosolic_p50-p65	& [1]PP2A-Bx
28	[0]ATM-P	& [1]nuclear_p50-p65-P	& [1]PP2A-Bx
29	[0]ATM-P	& [1]TAK1-P	& [1]PP2A-Bx
30	[0]Chk2-P	& [0]BARD1-BRCA1-P	& [1]PP2A-Bx
31	[0]MRN	& [0]Hsp90	& [0]Chk2-P
32	[0]Hsp90	& [0]Chk2-P	& [0]p53-PS15-PS20
33	[0]Chk2-P	& [1]PP2A-Bx	& [0]p53-PS15-PS20
34	[1]MDMX	& [0]Hsp90	& [0]Chk2-P
35	[1]MDM2	& [0]Chk2-P	& [0]Hsp90
36	[0]Hsp90	& [0]Chk2-P	& [1]PP2A-B55
37	[1]PP1	& [0]Chk2-P	& [0]Hsp90
38	[1]MDMX	& [0]Chk2-P	& [1]PP2A-Bx
39	[1]MDM2	& [0]Chk2-P	& [1]PP2A-Bx
40	[1]PP1	& [0]Chk2-P	& [1]PP2A-Bx
41	[0]MRN	& [1]PP2A-Bx	& [0]PIDD-RAIDD-caspase2
42	[1]Wip1	& [0]PIDD-RAIDD-caspase2	& [0]importin_alpha-1-beta-1
43	[1]Wip1	& [0]claspin-P	& [0]PIDD-RAIDD-caspase2
44	[1]Wip1	& [1]Cdc25A	& [0]PIDD-RAIDD-caspase2
45	[1]Wip1	& [1]Cdk2	& [0]PIDD-RAIDD-caspase2
46	[1]Wip1	& [2]IkappaBalpha	& [0]PIDD-RAIDD-caspase2
47	[1PP2A-Bx	& [1]PP2A-B55	& [0]PIDD-RAIDD-caspase2
48	[1]Wip1	& [1]PP2A-Bx	& [0]PIDD-RAIDD-caspase2
49	[0]FHIT	& [1]PP2A-Bx	& [0]BARD1-BRCA1-P
50	[0]MRN	& [1]PP2A-Bx	& [0]IkappaBalpha
51	[0]MRN	& [0]PIDD	& [1]PP2A-Bx
52	[0]MRN	& [0]FHIT	& [0]Hsp90
53	[0]MRN	& [0]Hsp90	& [1]PP2A-Bx
54	[0]MRN	& [1]p90-P	& [1]PP2A-Bx
55	[0]MRN	& [1]PP2A-Bx	& [2]IKK_complex-P
56	[0]MRN	& [1]MEK-P	& [1]PP2A-Bx
57	[0]MRN	& [1]ERK-P	& [1]PP2A-Bx
58	[0]MRN	& [1]PP2A-Bx	& [1]cytosolic_p50-p65
59	[0]MRN	& [1]PP2A-Bx	& [1]nuclear_p50-p65-P
60	[0]MRN	& [1]TAK1-P	& [1]PP2A-Bx
61	[1]Wip1	& [0]claspin-P	& [0]IkappaBalpha
62	[1]Wip1	& [1]Cdc25A	& [0]IkappaBalpha
63	[1]Wip1	& [1]Cdk2	& [0]IkappaBalpha
64	[1]PP2A-Bx	& [1]PP2A-B55	& [0]IkappaBalpha
65	[1]Wip1	& [1]PP2A-Bx	& [0]IkappaBalpha
66	[1]Wip1	& [0]PIDD	& [0]importin_alpha-1-beta-1
67	[1]Wip1	& [1]nuclear_p50-p65-P	& [0]importin_alpha-1-beta-1
68	[0]FHIT	& [0]Hsp90	& [0]p53-PS15-PS20
69	[0]FHIT	& [1]PP2A-Bx	& [0]p53-PS15-PS20
70	[1]Wip1	& [0]PIDD	& [0]claspin-P
71	[1]Wip1	& [0]PIDD	& [1]Cdc25A
72	[1]Wip1	& [0]PIDD	& [1]Cdk2
73	[1]Wip1	& [0]PIDD	& [2]IkappaBalpha
74	[0]PIDD	& [1]PP2A-Bx	& [1]PP2A-B55
75	[1]Wip1	& [0]PIDD	& [1]PP2A-Bx
76	[0]FHIT	& [1]MDMX	& [0]Hsp90
77	[0]FHIT	& [1]MDM2	& [0]Hsp90
78	[0]FHIT	& [0]Hsp90	& [1]PP2A-B55
79	[1]PP1	& [0]FHIT	& [0]Hsp90
80	[0]Hsp90	& [1]PP2A-Bx	& [1]PP2A-B55
81	[0]FHIT	& [1]MDMX	& [1]PP2A-Bx
82	[0]FHIT	& [1]MDM2	& [1]PP2A-Bx
83	[1]PP1	& [0]FHIT	& [1]PP2A-Bx
84	[1]Wip1	& [1]p90-P	& [0]claspin-P
85	[1]Wip1	& [0]claspin-P	& [2]IKK_complex-P
86	[1]Wip1	& [1]MEK-P	& [0]claspin-P
87	[1]Wip1	& [1]ERK-P	& [0]claspin-P
88	[1]Wip1	& [0]claspin-P	& [1]cytosolic_p50-p65
89	[1]Wip1	& [0]claspin-P	& [1]nuclear_p50-p65-P
90	[1]Wip1	& [1]p90-P	& [1]Cdc25A
91	[1]Wip1	& [1]Cdk2	& [1]p90-P
92	[1]p90-P	& [1]PP2A-Bx	& [1]PP2A-B55
93	[1]Wip1	& [1]p90-P	& [1]PP2A-Bx
94	[1]Wip1	& [1]Cdc25A	& [2]IKK_complex-P
95	[1]Wip1	& [1]Cdk2	& [2]IKK_complex-P
96	[1]PP2A-Bx	& [1]PP2A-B55	& [2]IKK_complex-P
97	[1]Wip1	& [1]PP2A-Bx	& [2]IKK_complex-P
98	[1]Wip1	& [1]MEK-P	& [1]Cdc25A
99	[1]Wip1	& [1]ERK-P	& [1]Cdc25A
100	[1]Wip1	& [1]Cdc25A	& [1]cytosolic_p50-p65
101	[1]Wip1	& [1]Cdc25A	& [1]nuclear_p50-p65-P
102	[1]Wip1	& [1]Cdk2	& [1]MEK-P
103	[1]Wip1	& [1]Cdk2	& [1]ERK-P
104	[1]Wip1	& [1]Cdk2	& [1]cytosolic_p50-p65
105	[1]Wip1	& [1]Cdk2	& [1]nuclear_p50-p65-P
106	[1]MEK-P	& [1]PP2A-Bx	& [1]PP2A-B55
107	[1]Wip1	& [1]MEK-P	& [1]PP2A-Bx
108	[1]ERK-P	& [1]PP2A-Bx	& [1]PP2A-B55
109	[1]Wip1	& [1]ERK-P	& [1]PP2A-Bx
110	[1]Wip1	& [2]IkappaBalpha	& [1]cytosolic_p50-p65
111	[1]Wip1	& [2]IkappaBalpha	& [1]nuclear_p50-p65-P
112	[1]PP2A-Bx	& [1]PP2A-B55	& [1]cytosolic_p50-p65
113	[1]Wip1	& [1]PP2A-Bx	& [1]cytosolic_p50-p65
114	[1]PP2A-Bx	& [1]PP2A-B55	& [1]nuclear_p50-p65-P
115	[1]TAK1-P	& [1]PP2A-Bx	& [1]PP2A-B55
116	[1]Wip1	& [1]PP2A-Bx	& [1]nuclear_p50-p65-P
117	[1]Wip1	& [1]TAK1-P	& [1]PP2A-Bx

It is important to note that the identified mutations might cause cell death by mitotic catastrophy only in response to severe DNA damage, as it is induced in cancer therapies. In contrast, in response to low levels of DNA damage, which is compatible with cell survival, the defects might promote tumorigenesis by enabling uncontrolled proliferation. Such limited DNA damage is caused permanently by cellular insults, like reactive oxygen species generated in metabolic processes [3].

## Conclusions

We presented a comprehensive logical model of the DDR including the dynamics of p53 and NF-κB regulation in human epithelial tumours. The large scale of the model and the implementation of posttranslational protein modifications allowed us to account for extensive crosstalk among signal transduction pathways. Our analyses enlightened the dynamics of the DDR and functional consequences of defects underlying cancerogenesis, but also hereditary genetic diseases. We identified candidate target proteins suitable for sensitization of epithelial tumours with different mutations to chemo- and radiotherapy, thus, our predicted target proteins provide a basis for follow up studies to demonstrate their therapeutic usefulness. Overall, the results reflect an approach to facilitate a holistic view on the DDR in health and disease. An important aim of further work is the inclusion of more quantitative data into the model. This would allow to recapitulate observations that lower levels of DNA damage predominantly induce temporary cell cycle arrest and DNA repair, whereas higher levels of DNA damage mainly cause apoptosis.

## Methods

### Data mining

For network assembly we screened the relevant literature through *NCBI/PubMed*. Large amounts of published experimental data were evaluated and only high quality data on causal relationships in human epithelial cells were used for modelling. By epithelial cells we refer to either epithelial cell lines in the sense of the *American Type Culture Collection*[72] or *ex vivo* epithelial cells. Information on intracellular localization of proteins was retrieved from [73] unless provided in the analyzed publications. Information on oncogenes and tumour suppressors were retrieved from [67,74].

### Interaction graph and discrete logical model

Some structural analyses were based on the representation of the structure underlying the studied model as a directed graph (interaction graph). Such a graph [17,75] consists of a set of nodes representing regulatory components (e.g. proteins, DNA damaging agents, ions), which are connected by arcs representing causal relationships. Signals are propagated from the start node to the end node of an arc. Activations are represented by arrows, whereas inhibitions are symbolized by T-shaped arcs. Each node is associated with a discrete logical state variable, which denotes the activity level of the corresponding regulatory component. The logical model is represented by a list of logical functions defining the target values of a component depending on the activity values of its regulators [18,76]. For combining logical variables in the logical functions we use a special notation of Boolean operators known as ‘sum of products’. Thereby we require the operators AND, OR, and NOT for describing any logical relationship [77]. Interactions are described by AND connections of (potentially negated) nodes. Each AND connection describes a sufficient condition for the activity of the target component. Additionally, a component may be activated by several distinct signalling events independently. This is expressed by a logical OR-connection.

The implementation of the ‘sum of products’ notation allows the representation of the logical model as a logical interaction hypergraph [17,78]. In the logical interaction hypergraph, interactions are represented by hyperarcs. In principle, hyperarcs can connect an arbitrary number of start nodes (regulating components) with an arbitrary number of end notes (regulated components). This distinguishes hyerarcs from arcs, which connect only one start node with one end node. Hyperarcs therefore allow the representation of logical AND-connections between nodes. In our network, each hyperarc points into only one end node. Additionally, a species may be activated by several distinct signalling events independently. Distinct hyperarcs pointing into the same end node represent logical OR connections. For simplicity, we also refer to hyperarcs if an interaction has only one start node. The logical interaction hypergraph was constructed using the software *CellDesigner 4.2*[79] and subsequently exported to the *MATLAB* (The MathWorks, Natick, MA, USA) package *CellNetAnalyzer 7.0*[80] for analyses (Additional file 2). In *CellNetAnalyzer*, the interaction graph underlying a given logical interaction hypergraph can be generated by splitting each hyperarc into its constituent arcs [80].

### Structural analyses

Based on the interaction graph the dependency matrix was calculated in *CellNetAnalyzer*. This matrix reveals functional interdependencies between each pair of species, e.g., it reveals whether a species *i* is a strong activator (i.e., there are only positive paths, and no species in these paths is regulated by a negative FL), a weak activator (i.e., there are only positive paths, and at least one species in these paths is regulated by a negative FL), a strong inhibitor (i.e., there are only negative paths, and no species in these paths is regulated by a negative FL), a weak inhibitor (i.e., there are only negative paths, and at least one species in these paths is regulated by a negative FL), or an ambivalent factor (i.e, positive and negative paths to the selected species exist) for another species *j*. This feature facilitates qualitative predictions of the effects of perturbations or knockout experiments [17,80].

FFLs and FLs were identified with the JAVA application *MAVisto V 2.7.0*[81] on the basis of the interaction graph underlying the logical model (Additional file 2). Negative FLs are a necessary condition for stable oscillations or homeostasis, whereas positive FLs are necessary for multistability [50,82]. The appearance of such dynamical behaviours further requires the loop to be functional. The functionality context of a feedback loop is defined as a set of constraints on the values of the external regulators of that loop [83]. The functionality context of each feedback loop in the logical model was identified on the basis of the logical model with the JAVA tool *GINsim 2.4 alpha*[84].

By computing logical steady states (LSS) of the logical network upon definition of a time scale value with *CellNetAnalyzer*[17] we studied the qualitative effects of input stimuli on downstream signalling events and on the outputs. The qualitative effects of loss of function mutations and inhibitions were studied by computing LSS after setting the activity levels of the relevant protein to ‘0’. Correspondingly, for studying the qualitative effects of constitutive activities, the activity level of the relevant protein was set to its highest possible value (‘1’ or ‘2’, respectively).

The calculation of LSS also provides the basis for calculations of minimal intervention sets with *CellNetAnalyzer* on the basis of the logical model [80,85]. These are minimal sets of regulatory components that are to be removed (by knockout, knockdown or inhibition) or to be added (by activation) to achieve a certain intervention goal. The maximum cardinality (maximum number of interventions allowed) of minimal intervention sets was set to 3.

### Dynamical analyses

Given a logical model and starting from an initial state of the network, consecutive states of the network can be computed. This is done by updating the activities of all components according to the logical functions. The computed dynamical behaviour of the network can be depicted in a state transition graph [84,86]. Each node in this graph represents a state of the network, i.e. a vector with its vector components representing the activity levels of all network components. The nodes are connected by arcs denoting possible state transitions. Usually, the reaction rates of the model interactions are unknown. Then, there are two basic strategies for dynamical analyses: synchronous and asynchronous updating [55]. In the first case, all activity levels are updated simultaneously. As each state can have at most one successor, the calculation of the state transition graph is quite simple, making it feasible even for large networks. Synchronous updating is based on the assumption that all components make a transition at the same time. This is unrealistic and can result in spurious dynamic behavior [87]. The second, more common strategy is to update only the activity level of one component at a time. The resulting state transition graph captures all possible state transitions, but is bigger than in the synchronous case. Accordingly, the state transition graph is more complex to model and analyse. We therefore restricted the computation of the state transition graph by applying an updating scheme with priority classes [88]. State transitions increasing a components activity are distinguished from state transitions decreasing its activity and were associated to priority classes with different ranks. The ranks were assigned to the priority classes according to the temporal order of interactions *in vivo*. At any state of the network, among all concurrent state transitions, only those of the class with the highest rank are triggered. As the temporal order of transitions belonging to the same priority class is unknown, we chose an asynchronous updating scheme for transitions belonging to the same class. Since the state space of a discrete logical network is finite, the system finally enters a LSS or a cycle of recurring states, called cyclic attractor [18]. Cyclic attractors are classified into simple loops and complex loops [55]. The former are cycles of network states such that each state can have exactly one successor state, whereas the latter are composed of overlapping simple loops. Dynamical analyses of the logical model were performed with *GINsim*.

### Network reduction

Dynamical analyses of large networks can be very challenging since the size of the state transition graph increases exponentially with network size. We therefore reduced the full model prior to dynamical analyses by removing components in iterative steps. In each of these steps, a component is removed by linking its regulators directly to its target components. Accordingly, the logical functions are properly rewritten. For instance, the cascade, MEK-P → ERK-P → p90-P’ can be reduced by removing the component ERK-P. This results in a reduced cascade, in which MEK-P activates p90-P directly. In the course of the model reduction, a FL can be reduced at most to its minimal form, an autoregulation. Autoregulated is a component that can either activate or inhibit itself. In the interaction graph autoregulation is indicated by a self loop, i.e., an arc with the start node and the end node representing the same component. By exclusion of autoregulated components from the reduction process, loss of feedback loops and attractors was avoided [54]. Model reduction was performed with *GINsim*.

### Model validation

The model was validated on the basis of published experimental studies.

## Competing interests

The authors declare that they have no competing interests.

## Authors' contributions

RP constructed the network model and designed the analysis. RP and MN drafted and wrote the manuscript. MN conceived the study and supervised the analysis. All authors have read and approved the final version of the manuscript.

## Supplementary Material

Additional file 1 **Table S1:** Logical functions of the model, **Table S2:** Logical steady states of the full model variants, **Table S3:** Excluded targets from search for putative therapeutic targets (see Table 3 of the main text), **Table S4:** Excluded targets from search for molecular defects putatively contributing to carcinogenesis (see Table 5 of the main text). Click here for file

Additional file 2 **The* CellDesigner *file corresponding to the logical interaction hypergraph shown in Figure **1**, the logical network in the* CellNetAnalyzer *and* GINsim *formats and the interaction graph in* MAVisto *format.** Click here for file
