# Supplementary material for: Dynamics of p53 and NF-κB regulation in response to DNA damage and identification of target proteins suitable for therapeutic intervention
Source: BMC Syst Biol. 2012 Sep 15;6:125. doi: 10.1186/1752-0509-6-125 (PMC3473366; doi:10.1186/1752-0509-6-125)
Supplement: Additional file 1 — Table S1: Logical functions of the model, Table S2: Logical steady states of the full model variants, Table S3: Excluded targets from search for putative therapeutic targets (see Table 3 of the main text), Table S4: Excluded targets from search for molecular defects putatively contributing to carcinogenesis (see Table 5 of the main text). [file 1752-0509-6-125-S1.pdf]

Supplementary Table 1 Logical functions of the model

Logical function	Time scale value	Explanation and references
1. camptothecin · topoisomeraseI → DNA SSBs	1	Camptothecin causes DNA single-strand breaks (SSBs) by inhibiting topoisomerase I (Pommier 2006).
2. SN38 · topoisomeraseI → DNA SSBs	1	SN38 causes SSBs by inhibiting topoisomerase I (Pommier 2006).
3. bleomycin → DNA DSBs early	1	Bleomycin causes DNA double-strand breaks (DSBs) (Povirk et al 1996). In the model, there are two components representing DSBs: 'DNA DSBs early' designates DSBs in the early phase of the DNA damage response (time scale value '1'), when DSBs have functions partially different from that in the later phase of the DNA damage response (time scale values '2' and '3') ('DNA DSBs late').
4. HU → DNA DSBs early	1	Hydroxyurea causes DSBs by blocking replication fork progression (Feng et al 2009, Saintigny et al 2001).
5. IR → DNA DSBs early	1	Ionizing radiation causes mainly DSBs by causing blockade of replication fork progression (Ward et al 1988, Löbrich et al 1995).
6. daunomycin · topoisomeraseII → DNA DSBs early	1	Daunomycin (daunorubicin) causes DSBs by inhibiting topoisomerase II (Andoh and Ishida 1998).
7. doxorubicin · topoisomeraseII → DNA DSBs early	1	Doxorubicin (adriamycin) causes DSBs by inhibiting topoisomerase II (De Beer et al 2001).
8. VP16 · topoisomeraseII → DNA DSBs early	1	VP16 (etoposide) causes DSBs primarily by inhibiting topoisomerase II (Baldwin and Osheroff 2005).
9. DNA SSBs → DNA DSBs early	1	During replication, SSBs can be transformed into DSBs (Strumberg et al 2000, Jackson and Bartek 2009).
10. DNA DSBs early → DNA DSBs late	2	DSBs in the early phase of the DNA damage response have roles different from that in the later phase.
11. DNA DSBs early → Ku	1	The Ku dimer becomes activated by binding to DSBs sites (Dong et al 2010).
12. Ku · PP5 → PARP1-PAR	1	The Ku dimer stimulates poly-ADP-ribosylation of PARP1; this depends on dephosphorylation of PARP1 by PP5 (Dong et al 2010).
13. !DNA SSBs · !DNA DSBs early → PP2A-Bx	1	PP2A-Bx ("Bx" designates the unknown identity of subunit B of the complex) is active in the absence of DNA damage, but inactive in the early phase of the response to SSBs or DSBs (Feng et al 2009, Leung-Pineda al 2006); compare logical function 77.
14. DNA SSBs · TopBP1 · !PP2A-Bx · PP5 → RPA-P-ATR-ATRIP-P	1	RPA and the ATR-ATRIP complexes bind to SSBs sites (Zou and Elledge 2003, Zhang et al 2005); this binding, as well as ATR kinase activity depends on catalytic activity of PP5 (Zhang et al 2005), ATR activation depends on TopBP1 (Liu et al 2006, Blackford et al 2010); in the late phase of the DNA damage response, PP2A-Bx inactivates the RPA-P-ATR-ATRIP-P complex by dephosphorylating RPA at threonine 21 and serine 33 (Feng et al 2009).
15. DNA SSBs → Cdc7	1	Cdc7 becomes activated in response to SSBs (Kim et al 2008).
16. DNA DSBs early → Cdc7	1	Cdc7 becomes activated in response to DSBs (Kim et al 2008).
17. nuclear c-Rel · Cdc7 → claspin-P	1	c-Rel expression is not induced by DNA damage, but constituitively active (Xu et al 2008, Radiation Genes Database); c-Rel in turn mediates constitutive expression of claspin (Kenneth et al 2010); Cdc7 actviates claspin by phosphorylation (Kim et al 2008).
18. RPA-P-ATR-ATRIP-P · claspin-P · Timeless-Tipin · !PP2A-Bx · Hsp90 → Chk1-P	1	ATR phosphorylates Chk1 at serines 296, 317 and 345, this depends on claspin (Kim et al 2008, Adams et al 2006, Zhao et al 2001, Petermann et al 2008, Pedram et al 2009) and on the Timeless-Tipin complex (Kemp et al 2010); PP2A-Bx deactivates Chk1 by dephosphorylating at least serines 317 and 345 (Leung-Pineda et al 2006); Hsp90 stabilizes Chk1 (Arlander et al 2003, Tse et al 2009).
19. RPA-P-ATR-ATRIP-P · FHIT · !Wip1 → Chk2-P	1	ATR phosphorylates Chk2 at threonine 68 (Wang et al 2006, Helt et al 2005), this depends on FHIT (Yutori et al 2008), Wip1 deactivates Chk2 by dephosphorylating it at threonine 68 (Fujimoto et al 2006).
20. 2 !ATM-P · !RPA-P-ATR-ATRIP-P · !Chk1-P · !Chk2-P → PP1	1	PP1 dephosphosphorylates p53 constitutively, thereby reducing its transcriptional activity; thus, kinases phosphorylating p53 in the early DNA damage response temporarily outcompete phosphatase PP1 (Li et al 2006a).
21. DNA DSBs early · MRN · PP5 · PP2A-B55 · !Wip1 → ATM-P<br><br>PP2A-B55 in this function is only included in the *GINsim* model, but not in the *CNA* model, because the algorithms function differently. The inclusion of PP2A-B55 allows for basal activation of ATM, leading to inhibition of PP2A-B55, as defined by logical function 22.	1	The MRN complex binds to DSBs sites and facilitates autophosphorylation of ATM at serine 1981 (Bakkenist and Kastan 2003, Dupré et al 2006, Robinson et al 2007), this is promoted by PP5 (Ali et al 2004). The main phosphatase dephosphorylating ATM at serine 1981 is Wip1 (Shreeram et al 2006).<br>Also in response to DSBs, the ATM-antagonizing phosphatase complex PP2A-B55 (PP2A complex including the subunit B55) becomes inactivated by dissociation; this is dependent on ATM kinase activity (logical function 22). This results in increased ATM activity. Thus, ATM and PP2A-B55 antagonize each other (Guo et al 2002, Goodarzi et al 2004, Freeman and Monteiro 2010). That issue might be explained by DSBs-induced low activity of ATM (activity level '1' in the logical model, described by logical function 21), which in turn is sufficient to inactivate PP2A-B55 (logical function 22). Now, the combination of PP2A-B55 inactivity and presence of DSBs might trigger full activation of ATM (i.e., ATM gains activity level '2' in the model, as described by logical function 23). Later (at time scale value 3); well before DNA damage is fixed by repair mechanisms, PP2A-B55 again becomes active (Guo et al 2002, Goodarzi et al 2004). In the model, this reactivation of PP2A-B55 in the late DNA damage resoponse is a consequence of Wip1-mediated downgegulation of the PP2A-B55-antagonizer ATM-P (logical functions 21 and 23).
22. !ATM-P → PP2A-B55	1	
23. DNA DSBs early · MRN · PP5 · !PP2A-B55 · !Wip1 → 2 ATM-P	1	
24. 2 ATM-P · MRN → MRN-P	1	ATM-P phosphorylates the MRN complex subunit Nbs1 at serine 343 (Lim et al 2000, Kurz et al 2004).
25. 2 ATM-P · FHIT · !Wip1 → Chk2-P	1	ATM-P phosphorylates Chk2 at threonine 68 (Melchionna et a 2000); this depends on FHIT (Yutori et al 2008); Wip1 dephosphorylates Chk2 at threonine 68 (Fujimoto et al 2006).
26. 2 ATM-P → c-Abl-P	1	ATM-P phosphorylates c-Abl (Baskaran et al 1997, Foray et al 2002,

		Waning et al 2011).
27. !c-Abl-P → MDM2	1	c-Abl-P mediates phosphorylation of MDM2 at serine 397, leading to ubiquitinylation-mediated proteolysis of MDM2 (Goldberg et al 2002, Waning et al 2011).
28. 2 !ATM-P · !Chk2-P · !MDM2 → MDMX	1	Chk2-P phosphorylates MDMX at serines 342 and 367, ATM-P phosphorylates MDMX at serine 403; both events lead to ubiquitinylation-mediated proteolysis of MDMX (Chen et al 2005, Pan et al 2003, Pereg et al 2005).<br>Independent of these events, the E3 ligase MDM2 polyubiquitinylates MDMX, leading to its proteolysis (Chen et al 2005, Pan et al 2003, Pereg et al 2005).
29. RPA-P-ATR-ATRIP-P · !MDMX · !MDM2 · !PP1 · PML → p53-P	1	ATR phosphorylates p53 at serine 15 (Lakin et al 1999, Zou and Elledge 2003).<br>MDM2 and MDMX polyubiquitinylate p53, leading to its proteolysis (Chen et al 2005, Goldberg et al 2002, Brooks et al 2007).<br>PP1 dephosphosphorylates p53 constitutively, thereby reducing transcriptional activity of p53; thus, phosphorylation of p53 in response to DNA damage temporarily outweighs its dephosphorylation (Li et al 2006a).<br>PML stabilizes p53 (Bao-Lei et al 2006).
30. 2 ATM-P → BARD1-BRCA1-P	1	ATM-P phosphorylates serines 1387, 1423 and 1524 of BRCA1; BARD1 stabilizes BRCA1 (Fabbro et al 2004, Cortez et al 1999, Gatei et al 2000).
31. 2 ATM-P · !MDMX · !MDM2 · BARD1-BRCA1-P · !PP1 · PML → p53-P	1	ATM-P phosphorylates p53 at serine 15, this depends on BARD1-BRCA1-P (Bakkenist and Kastan 2003, Fabbro et al 2004, Li et al 2006a).<br>See logical function 29 for effects of MDM2, MDMX, PML, and PP1 on p53.
32. Chk1-P · !MDMX · !MDM2 · !PP1 · PML → p53-P	1	Chk1-P phosphorylates p53 at serine 20 (Shieh et al 2000, Ou et al 2005).<br>See logical function 29 for effects of MDM2, MDMX, PML, and PP1 on p53.
33. Chk2-P · !MDMX · !MDM2 · !PP1 · PML → p53-P	1	Chk2-P mediates phosphorylation of p53 at serine 20 (Shieh et al 2000, Ou et al 2005).<br>See logical function 29 for effects of MDM2, MDMX, PML, and PP1 on p53.
34. !MDM2 → HIPK2	1	MDM2 polyubiquitinylates HIPK2, leading to its proteolysis; higher levels of DSBs lead to higher MDM2 degradation rates, such that HIPK2 becomes more stable (Rinaldo et al 2007, Dauth et al 2007). See logical function 27 for MDM2 degardation; the increase in degradation as a result of increased DNA damage is not captured by the model:
35. p53-P · HIPK2 → p53-P-PS46	1	HIPK2 phosphorylates already transription activing (and thus, phosphorylated) p53 at serine 46 (Rinaldo et al 2007, Dauth et al 2007).
36. 2 ATM-P · TopBP1 · !PP2A-Bx · PP5 → RPA-P-ATR-ATRIP-P	1	ATM-P recruits RPA-P-ATR-ATRIP to DSBs sites (Robison et al 2007, Zou and Elledge 2003, Adams et al 2006), the ATM-dependence of RPA–ATR-ATRIP recruitment to DSBs sites is also shown in fibroblasts (Jazayeri et al 2006, Reinhardt et al 2007). For TopBP1, PP2A-Bx, and PP5 see logical function 14.
37. Chk2-P → E2F-1-P	1	Chk2-P stabilizes E2F-1 by phosphoryling it at serine 364 (Stevens et al 2003).
38. !Chk1-P · !Chk2-P → Cdc25A	1	Chk1-P and Chk2-P phosphorylate Cdc25A at serines 76 and 123, respectively, leading to ubiquitinylation-mediated proteolysis of Cdc25A; Cdc25A is only stable only in absence of both, Chk1-P, and Chk2-P (Falck et al 2001, Jin et al 2008).
39. Cdc25A → Cdk2	1	Cdc25A activates Cdk2 by dephosphorylating it at tyrosine residues (Blomberg and Hoffmann 1999, Falck et al 2001).
40. Chk2-P → Cdc25C-P	1	Chk2-P phosphorylates Cdc25C at serine 216 (Matsuoka et al 1998, Pedram et al 2003).
41. DNA DSBs early · !DNA DSBs late → Cdt2-DDB1	1	DSBs trigger transient activation of the complex made of Cdt2 and DDB1. At time scale value 2, Cdt2-DDB1 is inactive (Stuart et al 2009).
42. DNA DSBs early · Hsp90 → PIDD	1	DSBs trigger activation of PIDD by autocatalytic cleavage, this depends on Hsp90 (α or β ?); activation of PIDD by DSBs in human epithelial cells is at least within the first hours after induction of DNA strand breaks independent of transactivation by p53 (Cuenin et al 2008, Tinel et al 2007, Tinel et al 2010).
43. PIDD → PIDD-RAIDD-caspase2	2	PIDD binds via RAIDD to caspase-2, which becomes activated. This occurs later than binding of PIDD to RIP1 and NEMO (logical function 44) (Cuenin et al 2008, Janssens et al 2005, Tinel and Tschopp 2004).
44. PIDD · NEMO → PIDD-RIP1-NEMO-PIASy	1	PIDD and NEMO (not bound to the IKK complex, S. Miyamoto, pers. communication) enter the nucleus and form a complex with RIP1 and PIASy (Tinel et al 2007, Janssens et al 2005, Mabb et al 2006, Wu et al 2006); a role of RIP1 in DNA DSBs-iduced NF-κB activation has additionally been shown in MEFs (Hur et al 2003).
45. PIDD-RIP1-NEMO-PIASy · !SENP2 → PIDD-RIP1-NEMO-S	1	PIASy sumoylates (SUMO-1) NEMO within the PIDD-RIP1-NEMO complex (Janssens et al 2005, Mabb et al 2006, Yang et al 2011a).<br>SENP2 desumoylates NEMO (Lee et al 2011).
46. 2 ATM-P · PIDD-RIP1-NEMO-S → ATM-P-RIP1-NEMO-P	1	ATM-P phosphorylates sumoylated NEMO at serine 85 (Huang et al 2003, Wu et al 2006). RIP1 is bound to ATM at least until TAK1 becomes phosphorylated in the cytosol (logical function 51) (Yang et al 2011a).
47. ATM-P-RIP1-NEMO-P · cIAP1 → nuclear ATM-P-RIP1-NEMO-Ub	1	Phosphorylated NEMO within the complex becomes monoubiquitinylated at lysine 277 and lysine 309 by cIAP1 (Huang et al 2003, Wu et al 2006, Jin et al 2009).
48. nuclear ATM-P-RIP1-NEMO-Ub · Ca^{2+} → cytosolic ATM-P-RIP1-NEMO-Ub	1	Export of ATM-P-RIP1-NEMO-Ub to the cytoplasm depends on Ca^{2+}, but there is nevertheless no increase in intracellular Ca^{2+} level within 6 h post DNA breakage (Wu et al 2006, Berchtold et al 2007, Gangadharan et al 2010).<br>RIP1 is bound to ATM at least until TAK1 becomes phosphorylated in the cytosol (logical function 51) (Yang et al 2011a).
49. cytosolic ATM-P-RIP1-NEMO-Ub · Ubc13 · XIAP → ATM-P-RIP1-NEMO-Ub-TRAF6-Ub-ELKS-Ub	1	TRAF6 becomes polyubiquitinylated dependent on ATM and Ubc13 (Hinz et al 2010); ELKS becomes polyubiquitinylated (K63-linked) dependent on ATM, Ubc13 and XIAP (Wu et al 2010); RIP1 is bound to ATM at least until TAK1 becomes phosphorylated in the cytosol (logical function 51) (Yang et al 2011a).<br>All proteins acting in this model between cytosolic ATM and activation of the IKK complex form a complex (Mabb et al 2006, Wu et al 2006, Jin et al 2009, Stilmann et al 2009, Wu et al 2010, Hinz et al 2010).
50. ATM-P-RIP1-NEMO-Ub-TRAF6-Ub-ELKS-Ub · LUBAC → ATM-P-RIP1-NEMO-Ub-TRAF6-Ub-ELKS-Ub-TAB	1	RIP1 is bound to ATM at least until TAK1 becomes phosphorylated in the cytosol (logical function 51) (Yang et al 2011a).

		All proteins acting in this model between cytosolic ATM and activation of the IKK complex form a complex (Mabb et al 2006, Wu et al 2006, Jin et al 2009, Stilmann et al 2009, Wu et al 2010, Hinz et al 2010).<br>Dependent on ATM-P, XIAP and ELKS-Ub, NEMO becomes linearly polyubiquitinylated at K285 and / or K309 by the LUBAC (SHARP-HOIP-HOIL-1) complex in the cytosol (Niu et al 2011).
51. ATM-P-RIP1-NEMO-Ub-TRAF6-Ub-ELKS-Ub-TAB → TAK1-P	1	Phosphorylation of TAK1 depends on ATM, ELKS, Ubc13, TRAF6, TAB (TAB1 or 2 ?) and XIAP, and linearly polyubiquitinylated NEMO (Wu et al 2010, Hinz et al 2010, Jin et al 2009, Niu et al 2011). RIP1 is bound to ATM at least until TAK1 becomes phosphorylated in the cytosol (logical function 51) (Yang et al 2011a).<br>All proteins acting in this model between cytosolic ATM and activation of the IKK complex form a complex (Mabb et al 2006, Wu et al 2006, Jin et al 2009, Stilmann et al 2009, Wu et al 2010, Hinz et al 2010).
52. RPA-P-ATR-ATRIP-P · TAK1-P → p38-P	1	Phosphorylations of p38α at T180 and Y182 depend on kinase activities of ATR and TAK1 (Yang et al 2011a, Cannell et al 2010). In response to DSBs, ATR kinase activity in turn depends on ATM-P (logical function 36) (see also Lafarga et al 2009); phosphorylated p38α is exported from the nucleus to the cytosol (Ben-Levy et al 1998).
53. p38-P → MK2-P	1	p38-P mediates phosphorylation of MK2 at threonine 334; this occurs also in presence of functional p53, as was shown in HEK293 cells (functional p53) (Yang et al 2011a, Cannell et al 2010).
54. p38-P · MK2-P → HuR	1	DSBs-induced phosphorylation of p38α leads to upregulation of Hur, this depends on MK2-P (Lafarga et al 2009).
55. MK2-P → miR-34c	1	MK2-P triggers expression of miR-34c; this occurs also in HEK293 cells (p53 wildtype) (Cannell et al 2010).
56. !miR-34c → c-Myc	1	miR-34c downregulates c-Myc expression ; this occurs also in HEK293 cells (p53 wildtype) (Cannell et al 2010).
57. !TAK1-P · !p90-P → PP1-CUEDC2	1	PP1-CUEDC2 dephosphorylates the IKK complex constitutively, thus, kinases phosphorylating the IKK complex in the DNA damage response (logical functions 62 and 63) temporarily outcompete phosphatase *PP1-CUEDC2* (Li et al 2008).
58. cytosolic ATM-P-RIP1-NEMO-Ub → MEK-P	1	cytosolic ATM-P-RIP1-NEMO-Ub mediates phosphorylation of MEK1/2, although earlier (which does not lead to activation of NF-κB, and are not included in the model) phosphorylations of MEK1/2 and ERK1/2 are independent of ATM (Panta et al 2004, Tang et al 2002, Li et al 2006b, Ahmed et al 2009).
59. MEK-P → ERK-P	1	MEK1/2 phosphorylates ERK1/2 (Panta et al 2004, Ahmed et al 2009).
60. ERK-P → p90-P	1	ERK1/2 mediates phosphorylation of $p90^{rsk1}$ (Panta et al 2004, Sturgill et al 1988).
61. IKK complex → IKK complex-P in *CNA*<br>!TAK1-P + !p90-P → IKK-P in *GINsim*	1	The basal phosphorylation of the IKK complex is sufficient for constitutive activation of c-Rel. c-Rel in turn mediates constitutive expression of claspin (Kenneth et al 2010, see also logical functions 67 and 70).
62. p90-P · !PP1-CUEDC2 · IKK complex → 2 IKK complex-P	1	$p90^{rsk1}$ mediates activation (level ‚2') of the IKK complex subunit IKKβ above its basal activity level (level '1') by phosphorylation (Panta et al 2004, Bottero et al 2001); $p90^{rsk1}$, like other IKKβ activating kinases, outcompetes PP1-CUEDC2 phosphatase from binding to the IKK complex (Li et al 2008).
63. TAK1-P · !PP1-CUEDC2 · IKK complex → 2 IKK complex-P	1	In response to DSBs, TAK1-P mediates activation (level '2') of the IKK complex subunit IKKβ above its basal activity level (level ‚1') by phosphorylation (Berchtold et al 2007, Li et al 1998, Jin et al 2009, Perkins 2007, Wu et al 2010, Hinz et al 2010, Bottero et al 2001); TAK1-P, like other IKKβ activating kinases, outcompetes PP1-CUEDC2 phosphatase from binding to the IKK complex (Li et al 2008).
64. !IKK complex-P → 2 IκBα<br>In *GINsim*, activity of IIκBα is not defined by means of a logical functions, but by defining all conditions under which IIκBα gains either activity level '0', '1'or '2', respectively.	1	IκBα is fully stable (activity level '2' in the model) only in absence of both, basal and stimulated activity of the IKK complex (activity level '0' in the model) (Li et al 1998, Strozyk et al 2006, Bottero et al 2001, DiDonato et al 1996).
65. !FKBP51 → IκBα<br>In *GINsim*, activity of IIκBα is not defined by means of a logical functions, but by defining all conditions under which IIκBα gains either activity level '0', '1'or '2', respectively.	1	FKBP51 is necessary for IKK-mediated degradation of IκBα (Romano et al 2004).
66. 2 !IKK complex-P → IκBα<br>In *GINsim*, activity of IIκBα is not defined by means of a logical functions, but by defining all conditions under which IIκBα gains either activity level '0', '1'or '2', respectively.	1	Inducers of SSBs and DSBs trigger phosphorylation of IκBα by IKK-P, leading to proteolysis of IκBα (Li et al 1998, Strozyk et al 2006, Bottero et al 2001, DiDonato et al 1996).
67. 2 !IκBα → cytosolic c-Rel	1	Constitutive low-level degradation of IκBα (driven by constitutive low activity of the IKK complex) releases c-Rel, which accordingly has a basal activity, leading to basal expression of claspin, as long as IκBα is not fully stabilized (i.e. gains activity level '2' in the model) (Hayden and Gosh 2008, Xu et al 2008, Kenneth et al 2010, RadiationGenes Database).
68. !IκBα → cytosolic p50-p50	1	Degradation of IκBα releases the NF-κB dimer p50-p50 (Hayden and Gosh 2008).
69. !IκBα → cytosolic p50-p65	1	Degradation of IκBα releases the NF-κB dimer p50-p65 (Hayden and Gosh 2008).
70. cytosolic c-Rel · importin α-1-β-1 → nuclear c-Rel	1	Released c-Rel binds prior to nuclear import to importin α-1 and importin β-1 (Hayden and Gosh 2008, Xu et al 2008).
71. cytosolic p50-p50 · importin α-1-β-1 → nuclear p50-p50	1	Released p50-p50 binds prior to nuclear import to importin α-1 and importin β-1 (Hayden and Gosh 2008, Xu et al 2008).
72. DNA DSBs early · IKKε → IKKε-P	1	DSBs trigger accumulation of IKKε in the nucleus, where IKKε becomes phosphorylated (Renner et al 2010).
73. IKKε-P · PML → PML-PS38	1	Inside the nucleus, IKKε-P phosphorylates PML at serine 38 (Renner et al 2010).
74. IKKε-P · PML-PS38 · TOPORS → IKKε-S-P	1	IKKε-P becomes sumoylated (SUMO-1) at lysine 231 by TOPORS; this depends on PML-PS38 (Renner et al 2010).
75. cytosolic p50-p65· importin α-1-β-1· IKKε-S-P → nuclear p50-p65-P	1	Released p50-p65 Upon binding to importin α-1 and importin β-1, p50-p65 becomes transported into the nucleus (Hayden and Gosh 2008, Xu et al 2008). IKKε-S-P mediates phosphorylation of p65 at serine 468, p65 additionally becomes phosphorylated at S536 (Renner et al 2010, Tapia et al 2007); both phosphorylations are known to activate the transactivation

		by p65 (Perkins 2006).
76. Chk2-P · PML → PML-PS117	1	Chk2-P phosphorylates PML at serine 117 (Yang et al 2002).
77. DNA DSBs late → PP2A-Bx	3	In the late DNA damage response (at time scale value 3) , PP2A-Bx becomes active again (Feng et al 2009, Leung-Pineda et al 2006). Since PP2A-Bx is not regulated by specific proteins in the model, it plays no role within the model, whether ATR and Chk1 are regulated by PP2A with the same B subunit (‘Bx’) or not.
78. !p53-P-PS46 → Bcl-3	1	p53-P (at least phosphorylated at serin 15) mediates destabilization of Bcl-3 (Rocha et al 2003); as this is probably a pro-apoptotic event, additional phosphorylation at serine 46 (the pro-apoptotic form of p53 in the model, see logical function 95) might be essential; due to the lack of direkt evidence supporting an anti-apoptotic role of Bcl-3 in response to SSBs or DSBs, Bcl-3 is not linked to apoptosis in the model.
79. !Bcl-3 → HDAC1	1	In the absence of Bcl-3, HDAC1 binds to chromatin-bound p52 dimers (Rocha et al 2003).
80. Bcl-3 · !HDAC1 → Bcl-3-p52-p52	1	Bcl-3 competes with HDAC1 for binding to the NF-κB dimer p52-p52; Bcl-3-p52-p52 binds to DNA and mediates transactivation of target genes (Rocha et al 2003).
81. p53-P · HuR · !Cdt2-DDB1 → p21	2	Early after induction of DSBs, Cdt2-DDB1 promotes polyubiquitinylation of p21, leading to its proteolysis; this process is independent of ATM (Stuart et al 2009); later (at time scale value 2), p53-P mediates expression of p21 (El-Deiry et al 1993, Han et al 2002, Vousden and Prives 2009), HuR stabilizes p21 mRNA by binding, thereby promoting G1/S cell cycle arrest (Lafarga et al 2009).
82. p53-P → Wip1	3	p53-P mediates expression of Wip1 during the late DNA damage response (Fiscella et al 1997).
83. p53-P-PS46 → HTRA2	3	Pro-apoptotic p53 (p53-P-PS46) mediates release of HTRA2 during the late DNA damage response (Bartling et al 2002, Jin et al 2003).
84. !HTRA2 → XIAP	1	HTRA2 (becoming active at time scale value 3, as described by logical function 83) binds to and mediates inactivation of XIAP (Srinivasula et al 2003, Martins et al 2002).
85. p53-P-PS46 → Diablo	3	Pro-apoptotic p53 (p53-P-PS46) mediates the increase of the level of cytosolic Smac/Diablo during the late DNA damage response (Yu et al 2007, Schuler and Green 2001, Bartling et al 2003).
86. !Diablo → cIAP1	1	Diablo (becoming active at time scale value 3, as described by logical function 85) inhibits cIAP1 (Du et al 2000, Dai et al 2008, Fandy et al 2008, Yang et al 2011b).
87. p53-P → MDM2	3	p53-P mediates expression of MDM2 during the late DNA damage response (Ard et al 2002, Phillips et al 2010, Lev-Bar et al 2000).
88. nuclear p50-p65-P → IκBα<br>In *GINsim*, activity of IIκBα is not defined by means of a logical functions, but by defining all conditions under which IIκBα gains either activity level ‘0’, ‘1’or ‘2’, respectively.	3	The NF-κB dimer p50-p65-P mediates expression of IκBα during the late response to many stimuli, including DNA strand breaks (Mabb et al 2006, Ma et al 2009, Hayden and Gosh 2008).
89. nuclear p50-p65-P → SENP2	3	The NF-κB dimer p50-p65-P mediates expression of SENP2 during the late DNA damage response (Lee et al 2011).
90. !c-Myc → CELL-CYCLE-ARREST	2	Downregulation of c-Myc leads to intra-S-phase cell cycle arrest (Cannell et al 2010).
91. p21 → CELL-CYCLE-ARREST	2	p21 mediates G1/S phase cell cycle arrest (Han et al 2002, Lafarga et al 2009).
92. !Cdk2 → CELL-CYCLE-ARREST	2	Loss of unphosphorylated Cdk2 leads to intra-S-phase cell cycle arrest (Blomberg and Hoffmann 1999, Falck et al 2001).
93. p53-P → CELL-CYCLE-ARREST	2	Phosphorylations of p53 at serine 15 and / or serine 20 trigger cell cycle arresting functions of p53, as long as it is not additionally phosphorylated at serine 46 (Vousden and Prives 2009, Jiang et al 2010).
94. Cdc25C-P → CELL-CYCLE-ARREST	2	Phosphorylation of Cdc25C at serine 216 causes G2/M-phase cell cycle arrest (Peng et al 1997, reviewed by Boutros et al 2006).
95. p53-P-PS46 · !nuclear_p50-p65-P → ONSET-OF-APOPTOSIS	2	Phosphorylation of p53 at serine 46 is known to mediate expression of pro-apoptotic target genes (Vousden and Prives 2009, Jiang et al 2010).<br>In most cases, NF-κB antagonizes apoptosis by transactivation of anti-apoptotic target genes in response to DSBs (Carson et al 2004, Wu and Miyamoto 2007, McCool and Miyamoto 2012).
96. E2F-1-P · !nuclear_p50-p65-P → ONSET-OF-APOPTOSIS	2	E2F-1-P promotes p53-independent (as well as p53-dependent) apoptosis (Stevens et al 2003, Dong et al 2003).<br>In most cases, NF-κB antagonizes apoptosis by transactivation of anti-apoptotic target genes in response to DSBs (Carson et al 2004, Wu and Miyamoto 2007, McCool and Miyamoto 2012).
97. PIDD-RAIDD-caspase2 · !nuclear_p50-p65-P → ONSET-OF-APOPTOSIS	2	Caspase 2 promotes apoptosis upon its activation within the PIDD/RAIDD/caspase2 complex (Tinel and Tschopp 2004, Tinel et al 2007, Cuenin et al 2008).<br>In most cases, NF-κB antagonizes apoptosis by transactivation of anti-apoptotic target genes in response to DSBs (Carson et al 2004, Wu and Miyamoto 2007, McCool and Miyamoto 2012).
98. PML-PS117 · !nuclear_p50-p65-P → ONSET-OF-APOPTOSIS	2	PML-PS117 mediates p53-independent apoptosis (Yang et al 2002).<br>In most cases, NF-κB antagonizes apoptosis by transactivation of anti-apoptotic target genes in response to DSBs (Carson et al 2004, Wu and Miyamoto 2007, McCool and Miyamoto 2012).

Symbols: ‘·’ logical AND, ‘!’ logical NOT

References supplementary Table 1

- Adams KE, Medhurst AL, Dart DA, Lakin ND. Recruitment of ATR to sites of ionising radiation-induced DNA damage requires ATM and components of the MRN protein complex. *Oncogene*. 2006;25:3894-3904.
- Ahmed KM, Nantajit D, Fan M, Murley JS, Grdina DJ, Li JJ. Coactivation of ATM/ERK/NF-kappaB in the low-dose radiation-induced radioadaptive response in human skin keratinocytes. *Free Radic Biol Med.* 2009;46:1543-50.
- Ali A, Zhang J, Bao S, Liu I, Otterness D, Dean NM, Abraham RT, Wang XF. Requirement of protein phosphatase 5 in DNA-damage-induced ATM activation. *Genes Dev.* 2004;18:249-254.
- Andoh T, Ishida R. Catalytic inhibitors of DNA topoisomerase II. *Biochim Biophys Acta*. 1998;1400:155-1571.
- Ard PG, Chatterjee C, Kunjibettu S, Adside LR, Gralinski LE, McMahon SB. Transcriptional regulation of the mdm2 oncogene by p53 requires TRRAP acetyltransferase complexes. *Mol Cell Biol.* 2002 ;22:5650-61.
- Arlander SJ, Eapen AK, Vroman BT, McDonald RJ, Toft DO, Karnitz LM. Hsp90 inhibition depletes Chk1 and sensitizes tumor cells to replication stress. *J Biol Chem.* 2003 ;278:52572-7.
- Bakkenist CJ, Kastan MB. DNA damage activates ATM through intermolecular autophosphorylation and dimer dissociation. *Nature*. 2003;421(6922):499-506.
- Baldwin EL, Osheroff N. Etoposide, topoisomerase II and cancer. *Curr Med Chem Anticancer Agents*. 2005;5:363-372.
- Bao-Lei T, Zhu-Zhong M, Yi S, Jun-Jie Q, Yan D, Hua L, Bin L, Guo-Wei Z, Zhi-Xian S. *J Cell Biochem.* 2006 Feb 15;97(3):561-71. Knocking down PML impairs p53 signaling transduction pathway and suppresses irradiation induced apoptosis in breast carcinoma cell MCF-7.
- Bartling B, Lewensohn R, Zhivotovsky B. Endogenously released Smac is insufficient to mediate cell death of human lung carcinoma in response to etoposide. Exp Cell Res. 2004 Aug 1;298:83-95.
- Baskaran R, Wood LD, Whitaker LL, et al. Ataxia telangiectasia mutant protein activates c-Abl tyrosine kinase in response to ionizing radiation. *Nature*. 1997;387:516-519.
- Ben-Levy R, Hooper S, Wilson R, Paterson HF, Marshall CJ. Nuclear export of the stress-activated protein kinase p38 mediated by its substrate MAPKAP kinase-2. *Curr Biol.* 1998 ;8:1049-57.
- Berchtold CM, Wu ZH, Huang TT, Miyamoto S. Calcium-dependent regulation of NEMO nuclear export in response to genotoxic stimuli. *Mol Cell Biol*. 2007;27:497-509.
- Blackford AN, Patel RN, Forrester NA, Theil K, Groitl P, Stewart GS, Taylor AM, Morgan IM, Dobner T, Grand RJ, Turnell AS. Adenovirus 12 E4orf6 inhibits ATR activation by promoting TOPBP1 degradation. *Proc Natl Acad Sci U S A.* 2010 ;107:12251-6.
- Blomberg I, Hoffmann I. Ectopic Expression of Cdc25A Accelerates the G1/S Transition and Leads to Premature Activation of Cyclin E- and Cyclin A-Dependent Kinases. *Mol and Cell Biol.*1999;19:6183-6194.
- Bottero V, Busuttil V, Loubat A, et al. Activation of Nuclear Factor κB through the IKK Complex by the Topoisomerase Poisons SN38 and Doxorubicin: A Brake to Apoptosis in HeLa Human Carcinoma Cells. *Cancer Res*. 2001;61:7785-7791.
- Boutros R, Dozier C, Ducommun B. The when and wheres of CDC25 phosphatases. Curr Opin Cell Biol. 2006;18:185-91.
- Brooks CL, Li M, Gu W. Mechanistic studies of MDM2-mediated ubiquitination in p53 regulation. *J Biol Chem*. 2007;282:22804-22815.
- Cannell IG, Kong YW, Johnston SJ, Chen ML, Collins HM, Dobbyn HC, Elia A, Kress TR, Dickens M, Clemens MJ, Heery DM, Gaestel M, Eilers M, Willis AE, Bushell M. p38 MAPK/MK2-mediated induction of miR-34c following DNA damage prevents Myc-dependent DNA replication. *Proc Natl Acad Sci U S A.* 2010;107:5375-80.
- Carson JP, Zhang N, Frampton GM, Gerry NP, Lenburg ME, Christman MF. Pharmacogenomic identification of targets for adjuvant therapy with the topoisomerase poison camptothecin. *Cancer Res.* 2004 64:2096-104.
- Chen L, Gilkes DM, Pan Y, Lane WS, Chen J. ATM and Chk2-dependent phosphorylation of MDMX contribute to p53 activation after DNA damage. *EMBO J*. 2005;3411-3422.
- Cortez D, Ang Y, Quin J, Elledge SJ. Requirement of ATM-Dependent Phosphorylation of Brca1 in the DNA Damage Response to Double-Strand Breaks. *Science.* 1999;286:1162-1166.
- Cuenin S, Tinel A, Janssens S, Tschopp J. p53-induced protein with a death domain (PIDD) isoforms differentially activate nuclear factor-kappaB and caspase-2 in response to genotoxic stress. *Oncogene.* 2008;27:387-396.
- Dai Y, Liu M, Tang W, DeSano J, Burstein E, Davis M, Pienta K, Lawrence T, Xu L. Molecularly targeted radiosensitization of human prostate cancer by modulating inhibitor of apoptosis. *Clin Cancer Res.* 2008 ;14:7701-10.
- Dauth I, Krüger J, Hofmann TG. Homeodomain-interacting protein kinase 2 is the ionizing radiation-activated p53 serine 46 kinase and is regulated by ATM. *Cancer Res.* 2007 67:2274-9. 140.
- De Beer EL, Bottone AE, Voest EE. Doxorubicin and mechanical performance of cardiac trabeculae after acute and chronic treatment: a review. *Eur. J. Pharmacol*. 2001;415:1-11.
- DiDonato J, Mercurio F, Rosette C, et al. Mapping of the inducible IkappaB phosphorylation sites that signal its ubiquitination and degradation. *Mol Cell Biol*. 1996;16:1295-1304.
- Dong F, Soubeyrand S, Haché RJ. Activation of PARP-1 in response to bleomycin depends on the Ku antigen and protein phosphatase 5. *Oncogene*. 2010 Jan 25. [Epub ahead of print]
- Dong YB, Yang HL, McMasters KM. E2F-1 overexpression sensitizes colorectal cancer cells to camptothecin. *Cancer Gene Ther*. 2003;10:168-178.
- Du C, Fang M, Li Y, Li L, Wang X. Smac, a mitochondrial protein that promotes cytochrome c-dependent caspase activation by eliminating IAP inhibition. *Cell.* 2000 Jul 7;10233-42.
- Dupré A, Boyer-Chatenet L, Gautier J. Two-step activation of ATM by DNA and the Mre11-Rad50-Nbs1 complex. *Nat Struct Mol Biol*. 2006;13:451-457.
- El-Deiry WS, Tokino T, Velculescu VE, et al. *WAF1*, a Potential Mediator of p53 Tumor Suppression. *Cell*. 1993;75:817-825.
- Fabbro M, Savage K, Hobson K, et al. BRCA1-BARD1 complexes are required for $p53^{Ser-15}$ phosphorylation and a G_1/S arrest following ionizing radiation-induced DNA damage. *J Biol Chem*. 2004;279(30):31251-31258.

- Falck J, Mailand N, Syljuasen RG, Bartek J, Lukas J. The ATM-Chk2-Cdc25A checkpoint pathway guards against radioresistant DNA synthesis. *Nature*. 2001;410(6830):842-847.
- Fandy TE, Shankar S, Srivastava RK. Smac/DIABLO enhances the therapeutic potential of chemotherapeutic drugs and irradiation, and sensitizes TRAIL-resistant breast cancer cells. *Mol Cancer.* 2008 Jun 30;7:60.
- Feng J, Wakeman T, Yong S, Wu X, Kornbluth S, Wang XF. Protein phosphatase 2A-dependent dephosphorylation of replication protein A is required for the repair of DNA breaks induced by replication stress. *Mol Cell Biol.* 2009 Nov;29(21):5696-709.
- Fiscella M, Zhang H, Fan S, Sakaguchi K, Shen S, Mercer WE, Vande Woude GF, O'Connor PM, Appella E. Wip1, a novel human protein phosphatase that is induced in response to ionizing radiation in a p53-dependent manner. *Proc Natl Acad Sci U S A.* 1997;94(12):6048-6053.
- Foray N, Marot D, Randrianarison V, et al. Constitutive association of BRCA1 and c-Abl and its ATM-dependent disruption after irradiation. 2002. *Mol Cell Biol*. 22(12):4020-4032.
- Freeman AK, Monteiro AN. Phosphatases in the cellular response to DNA damage. *Cell Commun Signal.* 2010 ;8:27.
- Fujimoto H, Onishi N, Kato N, Takekawa M, Xu XZ, Kosugi A, Kondo T, Imamura M, Oishi I, Yoda A, Minami Y. Regulation of the antioncogenic Chk2 kinase by the oncogenic Wip1 phosphatase. *Cell Death Differ.* 2006 ;13:1170-80.
- Gangadharan C, Thoh M, Manna SK. Late phase activation of nuclear transcription factor kappaB by doxorubicin is mediated by interleukin-8 and induction of apoptosis via FasL. *Breast Cancer Res Treat.* 2010 ;120:671-83.
- Gatei M*,* Scott SP, Filippovitch I, et al. Role for ATM in DNA Damage-induced Phosphorylation of BRCA1. *Cancer Res.* 2000;60(12):3299-3304.
- Goldberg Z, Sionov RV, Berger M, et al. Tyrosine phosphorylation of Mdm2 by c-Abl: implications for p53 regulation. *EMBO J*. 2002;21(14):3715-3727.
- Goodarzi AA, Jonnalagadda JC, Douglas P, Young D, Ye R, Moorhead GB, Lees-Miller SP, Khanna KK. Autophosphorylation of ataxia-telangiectasia mutated is regulated by protein phosphatase 2A. *EMBO J.* 2004 Nov 10;23(22):4451-61. Epub 2004 Oct 28.
- Guo CY, Brautigan DL, Larner JM. ATM-dependent dissociation of B55 regulatory subunit from nuclear PP2A-B55 in response to ionizing radiation. *J Biol Chem.* 2002 Feb 15;277(7):4839-44. E
- Han Z, Wei W, Dunaway S, et al. Role of p21 in Apoptosis and Senescence of Human Colon Cancer Cells Treated with Camptothecin*. J Biol Chem*. 2002;277(19):17154-17160.
- Hayden MS, Gosh S. Shared principles in NF-kappaB signaling. *Cell*. 2008;132(3):344-362.
- Hinz M, Stilmann M, Arslan SÇ, Khanna KK, Dittmar G, Scheidereit C. A cytoplasmic ATM-TRAF6-cIAP1 module links nuclear DNA damage signaling to ubiquitin-mediated NF-κB activation. *Mol Cell.* 2010 40:63-74.
- Huang TT, Wuerzberger-Davis SM, Wu ZH, Miyamoto S. Sequential modification of NEMO/IKKgamma by SUMO-1 and ubiquitin mediates NF-kappaB activation by genotoxic stress. *Cell*. 2003;115(5):565-576.
- Hur GM, Lewis J, Yang Q, Lin Y, Nakano H, Nedospasov S, Liu ZG. The death domain kinase RIP has an essential role in DNA damage-induced NF-kappa B activation. *Genes Dev.* 2003 Apr 1;17(7):873-82.
- Jackson SP, Bartek J. The DNA-damage response in human biology and disease. *Nature.* 2009 Oct 22;461(7267):1071-8. Review.
- Janssens S, Tinel A, Lippens S, Tschopp J. PIDD Mediates NF-κB Activation in Response to DNA Damage. *Cell.* 2005;123(6):1079-1092.
- Jazayeri A, Falck J, Lukas C, Bartek J, Smith GC, Lukas J, Jackson SP. ATM- and cell cycle-dependent regulation of ATR in response to DNA double-strand breaks. *Nat Cell Biol.* 2006 Jan;8(1):37-45. Jiang L, Sheikh MS, Huang Y. Decision Making by p53: Life versus Death. *Mol Cell Pharmacol.* 2010 Jan 1;2(2):69-77.
- Jin HS, Lee DH, Kim DH, Chung JH, Lee SJ, Lee TH. cIAP1, cIAP2, and XIAP act cooperatively via nonredundant pathways to regulate genotoxic stress-induced nuclear factor-kappaB activation*. Cancer Res.* 2009 Mar 1;69(5):1782-1791.
- Jin S, Kalkum M, Overholtzer M, Stoffel A, Chait BT, Levine AJ. CIAP1 and the serine protease HTRA2 are involved in a novel p53-dependent apoptosis pathway in mammals.Genes Dev. 2003 Feb 1;17(3):359-67.
- Jin J, Ang XL, Ye X, Livingstone M, Harper JW. Differential Roles for Checkpoint Kinases in DNA Damage-dependent Degradation of the Cdc25A Protein Phosphatase. *J Biol Chem*. 2008. 283(28):19322-19328.
- Kemp MG, Akan Z, Yilmaz S, Grillo M, Smith-Roe SL, Kang TH, Cordeiro-Stone M, Kaufmann WK, Abraham RT, Sancar A, Unsal-Kaçmaz K. Tipin-replication protein A interaction mediates Chk1 phosphorylation by ATR in response to genotoxic stress. *J Biol Chem.* 2010 May 28;285(22):16562-71.
- Kenneth NS, Mudie S, Rocha S. IKK and NF-kappaB-mediated regulation of Claspin impacts on ATR checkpoint function. *EMBO J.* 2010 Sep 1;29(17):2966-78. Kim JM, Kakusho N, Yamada M, Kanoh Y, Takemoto N, Masai H. Cdc7 kinase mediates Claspin phosphorylation in DNA replication checkpoint. *Oncogene.* 2008 May 29;27(24):3475-82.
- Kurz EU, Douglas P, Lees-Miller SP. Doxorubicin activates ATM-dependent phosphorylation of multiple downstream targets in part through the generation of reactive oxygen species. *J Biol Chem.* 2004 Dec 17;279(51):53272-81. Lafarga V, Cuadrado A, Lopez de Silanes I, Bengoechea R, Fernandez-Capetillo O, Nebreda AR. p38 Mitogen-activated protein kinase- and HuR-dependent stabilization of p21(Cip1) mRNA mediates the G(1)/S checkpoint. *Mol Cell Biol.* 2009 Aug;29(16):4341-51.
- Lakin ND, Hann SP, Jackson SP. The ataxia-telangiectasia related protein ATR mediates DNA-dependent phosphorylation of p53. *Oncogene*. 1999;18(27):3989-3995.
- Lee MH, Mabb AM, Gill GB, Yeh ET, Miyamoto S. NF-κB Induction of the SUMO Protease SENP2: A Negative Feedback Loop to Attenuate Cell Survival Response to Genotoxic Stress. *Mol Cell.* 2011 ;43:180-91.
- Leung-Pineda V, Ryan CE, Piwnica-Worms H. Phosphorylation of Chk1 by ATR is antagonized by a Chk1-regulated protein phosphatase 2A circuit. *Mol Cell Biol.* 2006 Oct;26(20):7529-38. Lev Bar-Or R, Maya R, Segel LA, Alon U, Levine AJ, Oren M. Generation of oscillations by the p53-Mdm2 feedback loop: a theoretical and experimental study. *Proc Natl Acad Sci U S A.* 2000 Oct 10;97(21):11250-5.
- Li DW, Liu JP, Schmid PC, Schlosser R, Feng H, Liu WB, Yan Q, Gong L, Sun SM, Deng M, Liu Y. Protein serine/threonine phosphatase-1 dephosphorylates p53 at Ser-15 and Ser-37 to modulate its transcriptional and apoptotic activities. *Oncogene.* 2006a May 18;25(21):3006-22.

- Li HY, Liu H, Wang CH, Zhang JY, Man JH, Gao YF, Zhang PJ, Li WH, Zhao J, Pan X, Zhou T, Gong WL, Li AL, Zhang XM. Deactivation of the kinase IKK by CUEDC2 through recruitment of the phosphatase PP1. *Nat Immunol.* 2008 May;9(5):533-41.
- Li N, Karin M. Ionizing radiation and short wavelength UV activate NF-kB through two distinct mechanisms. *Proc Natl Acad Sci USA*. 1998;95(22):13012-13017.
- Li Z, Hosoi Y, Cai K, Tanno Y, Matsumoto Y, Enomoto A, Morita A, Nakagawa K, Miyagawa K. Src tyrosine kinase inhibitor PP2 suppresses ERK1/2 activation and epidermal growth factor receptor transactivation by X-irradiation. *Biochem Biophys Res Commun.* 2006b Mar 10;341(2):363-8.
- Lim DS, Kim ST, Xu B, et al. ATM phosphorylates p95/nbs1 in an S-phase checkpoint pathway. *Nature*. 2000;404(6778):613-617.
- Liu S, Bekker-Jensen S, Mailand N, Lukas C, Bartek J, Lukas J. Claspin Operates Downstream of TopBP1 To Direct ATR Signaling towards Chk1 Activation. *Mol Cell Biol.* 2006 Aug;26(16):6056-64.
- Löbrich M, Rydberg B, Cooper PK. Repair of x-ray-induced DNA double-strand breaks in specific Not I restriction fragments in human fibroblasts: joining of correct and incorrect ends. *Proc Natl Acad Sci USA*. 1995;92(26):12050-12054.
- Ma Y, Wang M, Li N, Wu R, Wang X. Bleomycin-induced nuclear factor-kappaB activation in human bronchial epithelial cells involves the phosphorylation of glycogen synthase kinase 3beta. *Toxicol Lett*. 2009;187:194-200.
- Mabb AM, Wuerzberger-Davis SM, Miyamoto S. PIASy mediates NEMO sumoylation and NF-kappaB activation in response to genotoxic stress. *Nat Cell Biol.* 2006;8(9):986-993.
- Martins LM, Iaccarino I, Tenev T, Gschmeissner S, Totty NF, Lemoine NR, Savopoulos J, Gray CW, Creasy CL, Dingwall C, Downward J. The serine protease Omi/HtrA2 regulates apoptosis by binding XIAP through a reaper-like motif. *J Biol Chem.* 2002 Jan 4;277(1):439-44. Epub 2001 Oct 15.
- Matsuoka S, Huang M, Elledge SJ. Linkage of ATM to Cell Cycle Regulation by the Chk2 Protein Kinase. *Science.*1998;282(5395):1893-1897.
- McCool KW, Miyamoto S. DNA damage-dependent NF-κB activation: NEMO turns nuclear signaling inside out. *Immunol Rev.* 2012 Mar;246(1):311-26. doi: 10.1111/j.1600-065X.2012.01101.x.
- Melchionna R, Chen XB, Blasiani A, McGowan CH. Threonine 68 is required for radiation-induced phosphorylation and activation of Cds1. *Nat Cell Biol*. 2000;2(10):762-765.
- Niu J, Shi Y, Iwai K, Wu ZH. LUBAC regulates NF-κB activation upon genotoxic stress by promoting linear ubiquitination of NEMO. EMBO J. 2011 Aug 2;30(18):3741-53. doi: 10.1038/emboj.2011.264.
- Ou YH, Chung PH, Sun TP, Shieh SY. p53 C-terminal phosphorylation by CHK1 and CHK2 participates in the regulation of DNA-damage-induced C-terminal acetylation. *Mol Biol Cell.* 2005 ;16:1684-95.
- Pan Y, Chen J. MDM2 Promotes Ubiquitination and Degradation of MDMX. *Mol Cell Biol*. 2003;23:5113-5121.
- Panta GR, Kaur S, Cavin LG, et al. ATM and the catalytic subunit of DNA-dependent protein kinase activate NF-kappaB through a common MEK/extracellular signal-regulated kinase/p90(rsk) signaling pathway in response to distinct forms of DNA damage. *Mol Cell Biol*. 2004;24(5):1823-1835.
- Pedram A, Razandi M, Evinger AJ, Lee E, Levin ER. Estrogen inhibits ATR signaling to cell cycle checkpoints and DNA repair. *Mol Biol Cell.* 2009 Jul;20(14):3374-89. Epub 2009 May 28.
- Peng CY, Graves PR, Thoma RS, Wu Z, Shaw AS, Piwnica-Worms H. Mitotic and G2 checkpoint control: regulation of 14-3-3 protein binding by phosphorylation of Cdc25C on serine-216. *Science.* 1997 5;277:1501-5.
- Pereg Y, Shkedy D, de Graaf P, et al. Phosphorylation of Hdmx mediates its Hdm2- and ATM-dependent degradation in response to DNA damage. *Proc Natl Acad Sci U S A.* 2005;102(14):5056-5061. Epub 2005 May 23.
- Perkins ND. Integrating cell-signalling pathways with NF-kappaB and IKK function. *Nat Rev Mol Cell Biol.* 2007 ;8:49-62.
- Perkins ND. Post-translational modifications regulating the activity and function of the nuclear factor kappa B pathway. Oncogene. 2006 Oct 30;25(51):6717-30.
- Petermann E, Helleday T, Caldecott KW. Claspin promotes normal replication fork rates in human cells. *Mol Biol Cell.* 2008 Jun;19(6):2373-8.
- Phillips A, Teunisse A, Lam S, Lodder K, Darley M, Emaduddin M, Wolf A, Richter J, de Lange J, Verlaan-de Vries M, Lenos K, Böhnke A, Bartel F, Blaydes JP, Jochemsen AG. HDMX-L is expressed from a functional p53-responsive promoter in the first intron of the HDMX gene and participates in an autoregulatory feedback loop to control p53 activity. *J Biol Chem.* 2010 Sep 17;285(38):29111-27. Epub 2010 Jul 20.
- Pommier Y. Topoisomerase I inhibitors: camptothecins and beyond. *Nat Rev Cancer.* 2006; 6(10):789-802.
- Povirk LF. DNA damage and mutagenesis by radiomimetic DNA-cleaving agents: bleomycin,neocarzinostatin and other enediynes. *Mutat Res.* 1996;355(1-2):71-89.
- Radiation genes database: http://t.caspur.it/RadioGenes/
- Reinhardt HC, Aslanian AS, Lees JA, Yaffe MB. p53-deficient cells rely on ATM- and ATR-mediated checkpoint signaling through the p38MAPK/MK2 pathway for survival after DNA damage. *Cancer Cell.* 2007 Feb;11(2):175-89.
- Renner F, Dorta, RM, Schmitz L. SUMOylation-dependent localization of IKKepsilon in PML nuclear bodies is essential for protecting against DNA damage-triggered cell death. *Mol Cell.* 2010 Feb 26;37(4):503-15.
- Rinaldo C, Prodosmo A, Mancini F, Iacovelli S, Sacchi A, Moretti F, Soddu S. MDM2-Regulated Degradation of HIPK2 Prevents p53Ser46 Phosphorylation and DNA Damage-Induced Apoptosis. *Mol Cell.* 2007 Mar 9;25(5):739-50.
- Robison JG, Bissler JJ, Dixon K. Replication Protein A is Required for Etoposide-Induced Assembly of MRE11/RAD50/NBS1 Complex Repair Foci. *Cell Cycle*. 2007;6(19):2408-2416. Epub 2007 Jul 20.
- Rocha S, Martin AM, Meek DW, Perkins ND. p53 represses cyclin D1 transcription through down regulation of Bcl-3 and inducing increased association of the p52 NF-kappaB subunit with histone deacetylase 1. *Mol Cell Biol*. 2003;23(13):4713-4727.

- Romano MF, Avellino R, Petrella A, Bisogni R, Romano S, Venuta S. Rapamycin inhibits doxorubicin-induced NF-kappaB/Rel nuclear activity and enhances the apoptosis of melanoma cells. *Eur J Cancer.* 2004 Dec;40(18):2829-36.
- Saintigny Y, Delacôte F, Varès G, Petitot F, Lambert S, Averbeck D, Lopez BS. Characterization of homologous recombination induced by replication inhibition in mammalian cells. *EMBO J.* 2001 Jul 16;20(14):3861-70.
- Schouten, G. J. et al. IkBa is a target for the mitogen-activated 90 kDa ribosonal S6 kinse. *EMBO J.* 16, 3133-3144 (1997).
- Schuler M, Green DR. Mechanisms of p53-dependent apoptosis. *Biochem Soc Trans.* 2001 ;29:684-8.
- Shieh SY, Ahn J, Tamai K, et al. The human homologs of checkpoint kinases Chk1 and Cds1 (Chk2) phosphorylate p53 at multiple DNA damage-inducible sites. *Genes Dev*. 2000;14(3):289-300.
- Shreeram S, Demidov ON, Hee WK, Yamaguchi H, Onishi N, Kek C, Timofeev ON, Dudgeon C, Fornace AJ, Anderson CW, Minami Y, Appella E, Bulavin DV. Wip1 phosphatase modulates ATM-dependent signaling pathways. *Mol Cell.* 2006 Sep 1;23(5):757-64.
- Srinivasula SM, Gupta S, Datta P, Zhang Z, Hegde R, Cheong N, Fernandes-Alnemri T, Alnemri ES. Inhibitor of apoptosis proteins are substrates for the mitochondrial serine protease Omi/HtrA2. *J Biol Chem.* 2003 Aug 22;278(34):31469-72. Epub 2003 Jun 30.
- Stevens C, Smith L, La Thangue NB. Chk2 activates E2F-1 in response to DNA damage. *Nat Cell Biol.* 2003;5(5):401-409.
- Stilmann M, Hinz M, Arslan SC, Zimmer A, Schreiber V, Scheidereit C. A nuclear poly(ADP-ribose)-dependent signalosome confers DNA damage-induced IkB kinase activation. *Mol Cell.* 2009;36(3):365- 378.
- Strozyk E, Pöppelmann B, Schwarz T, Kulms D. Differential effects of NF-κB on apoptosis induced by DNA damage determines the final outcome. *Oncogene*. 2006;25(47):6239-6251. Epub 2006 May 15.
- Strumberg D, Pilon AA, Smith M, Hickey R, Malkas L, Pommier Y. Conversion of topoisomerase I cleavage complexes on the leading strand of ribosomal DNA into 5'-phosphorylated DNA double-strand breaks by replication runoff. *Mol Cell Biol.* 2000 Jun;20(11):3977-87.
- Stuart SA, Wang JY. Ionizing radiation induces ATM-independent degradation of p21Cip1 in transformed cells. *J Biol Chem.* 2009 ;284:15061-70.
- Sturgill TW, Ray LB, Erikson E, Maller JL. Insulin-stimulated MAP-2 kinase phosphorylates and activates ribosomal protein S6 kinase II. *Nature.* 1988 ;334:715-8.
- Tang D, Wu D, Hirao A, Lahti JM, Liu L, Mazza B, Kidd VJ, Mak TW, Ingram AJ. ERK activation mediates cell cycle arrest and apoptosis after DNA damage independently of p53. J Biol Chem. 2002;277:12710-7.
- Tapia MA, González-Navarrete I, Dalmases A, Bosch M, Rodriguez-Fanjul V, Rolfe M, Ross JS, Mezquita J, Mezquita C, Bachs O, Gascón P, Rojo F, Perona R, Rovira A, Albanell. J. Inhibition of the canonical IKK/NF kappa B pathway sensitizes human cancer cells to doxorubicin. *Cell Cycle.* 2007 ;6: 2284-92.
- Tinel A, Eckert MJ, Logette E, Lippens S, Janssens S, Jaccard B, Quadroni M, Tschopp J. Regulation of PIDD auto-proteolysis and activity by the molecular chaperone Hsp90. *Cell Death Differ.* 2010 Oct 22. [Epub]
- Tinel A, Janssens S, Lippens S, et al. Autoproteolysis of PIDD marks the bifurcation between pro-death caspase-2 and pro-survival NF-kappaB pathway. *EMBO J.* 2007;26:197-208.
- Tinel A, Tschopp J. The PIDDosome, a protein complex implicated in activation of caspase-2 in response to genotoxic stress. *Science.* 2004;304:843-846.
- Tse AN, Sheikh TN, Alan H, Chou TC, Schwartz GK. 90-kDa heat shock protein inhibition abrogates the topoisomerase I poison-induced G2/M checkpoint in p53-null tumor cells by depleting Chk1 and Wee1. *Mol Pharmacol.* 2009;75:124-33.
- Vousden KH, Prives C. Blinded by the Light: The Growing Complexity of p53. *Cell.* 2009 ;137:413-31.
- Waning DL, Lehman JA, Batuello CN, Mayo LD. c-Abl phosphorylation of Mdm2 facilitates Mdm2-Mdmx complex formatio*n. J Biol Chem.* 2011 ;286:216-22.
- Ward, J. F. DNA damage produced by ionizing radiation in mammalian cells: identities, mechanisms of formation, and reparability. *Prog. Nucleic Acid Res. Mol. Biol.* 35, 95–125 (1988).
- Wu ZH, Miyamoto S. Many faces of NF-κB signaling induced by genotoxic stress*. J Mol Med.* 2007;85:1187-1202.
- Wu ZH, Shi Y, Tibbetts RS, Miyamoto S. Molecular Linkage Between the Kinase ATM and NF-κB Signalling in Response to Genotoxic Stimuli. *Science.* 2006; 311:1141-1146.
- Wu ZH, Wong ET, Shi Y, Niu J, Chen Z, Miyamoto S, Tergaonkar V. ATM- and NEMO-dependent ELKS ubiquitination coordinates TAK1-mediated IKK activation in response to genotoxic stress. *Mol Cell.* 2010 ;40:75-86.
- Xu Y, Fang F, St Clair DK, Sompol P, Josson S, St Clair WH. SN52, a novel nuclear factor-kappaB inhibitor, blocks nuclear import of RelB:p52 dimer and sensitizes prostate cancer cells to ionizing radiation. *Mol Cancer Ther.* 2008;7:2367-2376.
- Yang J, McEachern D, Li W, Davis MA, Li H, Morgan MA, Bai L, Sebolt JT, Sun H, Lawrence TS, Wang S, Sun Y. Radiosensitization of head and neck squamous cell carcinoma by a SMAC-mimetic compound, SM-164, requires activation of caspases. *Mol Cancer Ther.* 2011b ;10:658-69.
- Yang S, Kuo C, Bisi JE, Kim MK. PML-dependent apoptosis after DNA damage is regulated by the checkpoint kinase hCds1/Chk2. *Nat Cell Biol.* 2002 Nov;4:865-70.
- Yang Y, Xia F, Hermance N, Mabb A, Simonson S, Morrissey S, Gandhi P, Munson M, Miyamoto S, Kelliher MA. A cytosolic ATM/NEMO/RIP1 complex recruits TAK1 to mediate the NF-{kappa}B and p38 MAP kinase/MAPKAP-2 responses to DNA damage. *Mol Cell Biol.* 2011a May 23.
- Yu J, Wang P, Ming L, Wood MA, Zhang L. SMAC/Diablo mediates the proapoptotic function of PUMA by regulating PUMA-induced mitochondrial events. *Oncogene.* 2007 ;26:4189-98.
- Yutori H, Semba S, Komori T, Yokozaki H. Restoration of fragile histidine triad expression restores Chk2 activity in response to ionizing radiation in oral squamous cell carcinoma cells. *Cancer Sci.* 2008 99:524-30.
- Zhang J, Bao S, Furumai R, Kucera KS, Ali A, Dean NM, Wang XF. Protein phosphatase 5 is required for ATR-mediated checkpoint activation. *Mol Cell Biol.* 2005 ;25:9910-9.
- Zhao H, Piwnica-Worms H. ATR-Mediated Checkpoint Pathways Regulate Phosphorylation and Activation of Human Chk1. *Mol Cell Biol*. 2001;21:4129-4139.

- Zou L, Elledge SJ. Sensing DNA Damage Through ATRIP Recognition of RPA-ssDNA Complexes. *Science*. 2003;300:1542-1548.

Supplementary Table 2

Regulatory component	Logical steady states				
	A	B	C	D	E
APOPTOSIS, ONSET OF	no logical steady state	no logical steady state	no logical steady state	0	1
ATM-P				2	0
ATM-P-RIP1-NEMO-P				0	1
ATM-P-RIP1-NEMO-Ub-TRAF6-Ub-ELKS-Ub				0	0
ATM-P-RIP1-NEMO-Ub-TRAF6-Ub-ELKS-Ub-TAB				0	0
BARD1-BRCA1				1	0
Bcl-3				1	1
Bcl-3-p52-p52				1	1
bleomycin				0	0
Ca^{2+}				1	1
c-Abl-P				1	0
camptothecin				0	0
Cdc25A				0	1
Cdc25C-P				1	0
Cdc7				1	1
Cdt2-DDB1				0	0
Cdk2				0	1
CELL-CYCLE-ARREST				1	1
Chk1-P				0	0
Chk2-P				1	0
cIAP1				1	1
claspin-P				1	1
c-Myc				1	1
cytosolic_ATM-P-RIP1-NEMO-Ub				0	0
cytosolic_c-Rel				1	1
cytosolic_p50-p50				0	0
cytosolic_p50-p65				0	0
daunomycin				0	0
Diablo				0	0
DNA_DSBs_early				1	1
DNA_DSBs_late				1	1
DNA_SSBs				0	0
doxorubicin				0	0
E2F-1-P				1	0
ERK-P				0	0
FHIT				1	1
FKBP51				1	1
HDAC1				0	0
HIPK2				1	0
Hsp90				1	1
HTRA2				0	0
HU				0	0
HuR				0	0
IκBα				1	1
IKK_complex-P				1	1
IKKε				1	1
IKKε-P				1	1
IKKε-S-P				1	1
importin_α-1_β-1				1	1
IR				1	1
Ku				1	1
LUBAC				0	0
MDM2				0	1
MDMX				0	0
MEK-P				0	0
miR-34c				0	0
MK2-P				0	0
MRN				1	1
MRN-P				1	0
NEMO				1	1
nuclear_ATM-P-RIP1-NEMO-Ub				0	0
nuclear_c-Rel				1	1

nuclear_p50-p50				1	0
nuclear_p50-p65-P				1	0
p21				0	0
p38-P				0	0
p53-P				0	1
p53-P-PS46				0	0
p90-P				0	0
PARP1-PAR				1	1
PIDD				1	1
PIDD-RAIDD-caspase2				1	1
PIDD-RIP1-NEMO-PIASy				1	1
PIDD-RIP1-NEMO-S				0	1
PML				1	1
PML-PS117				1	0
PML-PS38				1	1
PP1				0	1
PP1-CUEDC2				1	1
PP2A-B55				0	1
PP2A-Bx				1	1
PP5				1	1
RPA-P-ATR-ATRIP-P				0	0
SENP2				1	0
SN38				0	0
TAK1-P				0	0
Timeless-Tipin				1	1
TopBP1				1	1
topoisomeraseI				1	1
topoisomeraseII				1	1
TOPORS				1	1
Ubc13				1	1
VP16				0	0
Wip1				0	1
XIAP				1	1

Supplementary Table 3 Excluded targets from search (see Table 2)

Excluded targets	Reasons
ATRIP	no enzymatic activity (UniProtKB)
Ca^{2+}	has too many functions not captured by the model (Bootman et al 2009)
c-Abl	has too many functions not captured by the model (UniProtKB)
Cdc7	required for proliferation (UniProtKB)
cIAP1	has too many functions not captured by the model (UniProtKB)
claspin-P	no enzymatic activity (UniProtKB)
ELKS	no enzymatic activity (UniProtKB)
FHIT	substrate analog inhibitors of FHIT may actually promote its function in the DNA damage response (Varnum et al 2001, Saldivar et al 2010)
importin_α1_β1	has too many functions not captured by the model (UniProtKB)
miR-34c	microRNA, has thus no enzymatic activity
NEMO	no enzymatic activity (UniProtKB)
p38α	has too many functions not captured by the model (UniProtKB)
PIASy	has too many functions not captured by the model (UniProtKB)
PML	has too many functions not related to DNA damage response (UniProtKB)
RIP1	role of RP1 in the model independent of its kinase activity (Biton and Ashkenazi 2011)
RPA	required for proliferation (UniProtKB)
TAB proteins	no enzymatic activity (UniProtKB)
Timeless-Tipin	required for proliferation (UniProtKB)
TopBP1	required for proliferation (Mäkiniemi et al 2001)
TOPORS	promotes proliferation (UniProtKB)
Ubc13	required for proliferation (UniProtKB)

References Supplementary Table 3

- Biton S, Ashkenazi A. NEMO and RIP1 control cell fate in response to extensive DNA damage via TNF-α feedforward signaling. Cell. 2011 Apr 1;145(1):92-103.
- Bootman MD, Fearnley C, Smyrnias I, MacDonald F, Roderick HL. An update on nuclear calcium signalling. J Cell Sci. 2009 Jul 15;122(Pt 14):2337-50. Review.
- Mäkiniemi M, Hillukkala T, Tuusa J, Reini K, Vaara M, Huang D, Pospiech H, Majuri I, Westerling T, Mäkelä TP, Syväoja JE. BRCT domain-containing protein TopBP1 functions in DNA replication and damage response. J Biol Chem. 2001 Aug 10;276(32):30399-406.
- Saldivar JC, Shibata H, Huebner K. Pathology and biology associated with the fragile FHIT gene and gene product. J Cell Biochem. 2010 Apr 1;109(5):858-65. Review.
- *UniProtKB Protein Knowledgebase*: http://www.uniprot.org/uniprot/
- Varnum JM, Baraniak J, Kaczmarek R, Stec WJ, Brenner C. Di-, tri- and tetra-5'-O-phosphorothioadenosyl substituted polyols as inhibitors of Fhit: Importance of the alpha-beta bridging oxygen and beta phosphorus replacement. BMC Chem Biol. 2001;1(1):3.

Supplementary Table 4 Excluded targets from search (see Table 4)

Excluded activations	Reasons
ATM-P	tumor suppressor (Tumor Gene Family of Databases)
BARD1-BRCA1-P	BRCA1 is a tumor suppressor (Tumor Gene Family of Databases)
Chk2-P	tumor suppressor (Sherr et al 2004)
E2F1-P	tumor suppressor (Tumor Gene Family of Databases)
FHIT	tumor suppressor (Tumor Gene Family of Databases)
p53-P	tumor suppressor (Tumor Gene Family of Databases)

Excluded inactivations	Reasons
c-Abl-P	proto-oncogene (Tumor Gene Family of Databases)
Cdc7	overexpression associated with tumor growth (UniProtKB)
Chk1-P	necessary for proliferation (Petermann et al 2010)
c-Myc	proto-oncogene (Tumor Gene Family of Databases)
c-Rel	proto-oncogene (Tumor Gene Family of Databases)
MDM2	proto-oncogene (Tumor Gene Family of Databases)
PML	proto-oncogene (Tumor Gene Family of Databases)
PP1	necessary for proliferation (UniProtKB)
RPA-P-ATR-ATRIP-P	ATR and RPA are necessary for proliferation (Nam and Cortez 2011, UniProtKB)
Timeless-Tipin	necessary for proliferation (UniProtKB)
TopBP1	necessary for proliferation (UniProtKB)
topoisomerase I	proto-oncogene (UniProtKB)

References Supplementary Table 4

- Nam EA, Cortez D. ATR signalling: more than meeting at the fork. Biochem J. 15;436:527-36.
- Petermann E, Woodcock M, Helleday T. Chk1 promotes replication fork progression by controlling replication initiation. Proc Natl Acad Sci U S A. 2010 107:16090-5.
- Sherr CJ. Principles of tumor suppression. Cell. 2004;116:235-46.
- *The Tumor Gene Family of Databases*: http://www.tumor-gene.org/tgdf.html
- *UniProtKB Protein Knowledgebase*: http://www.uniprot.org/uniprot/
