# Supplementary figures and images for: Dynamics of p53 and NF-κB regulation in response to DNA damage and identification of target proteins suitable for therapeutic intervention
Source: BMC Syst Biol. 2012 Sep 15;6:125. doi: 10.1186/1752-0509-6-125 (PMC3473366; doi:10.1186/1752-0509-6-125)

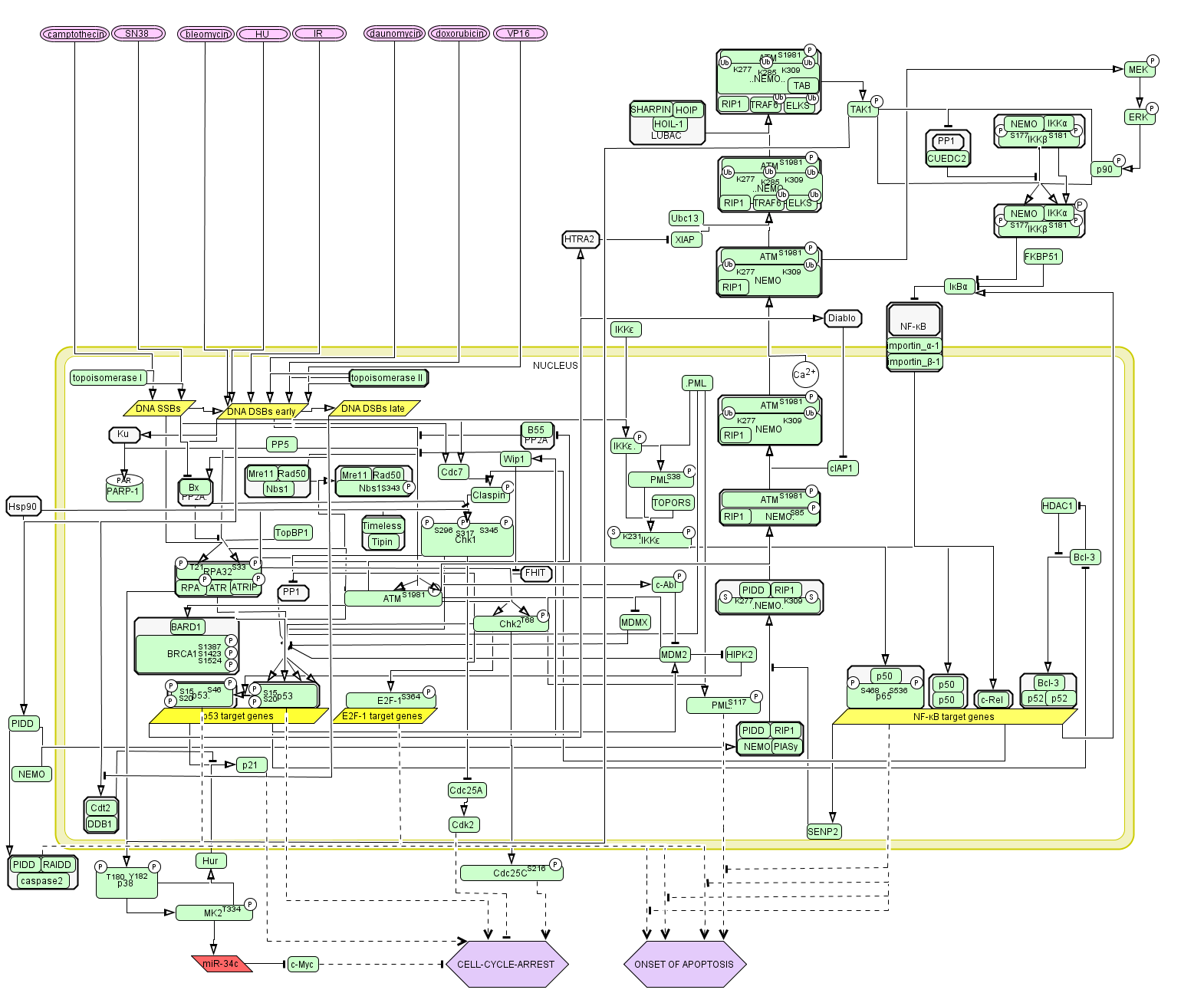

Supplement: Additional file 2 — The CellDesigner file corresponding to the logical interaction hypergraph shown in Figure 1, the logical network in the CellNetAnalyzer and GINsim formats and the interaction graph in MAVisto format. [file 1752-0509-6-125-S2.zip › Suppl_Folder_1/CNA-modell_DDR/CNA-network-graph.png]
